# First evidence of the link between internal and external structure of the human inner ear otolith system using 3D morphometric modeling

**DOI:** 10.1038/s41598-023-31235-1

**Published:** 2023-03-24

**Authors:** Christopher M. Smith, Ian S. Curthoys, Jeffrey T. Laitman

**Affiliations:** 1 Department of Anthropology, The Graduate Center, City University of New York, New York, NY 10016 USA; 2 Center for Anatomy and Functional Morphology, Icahn School of Medicine at Mount Sinai, New York, NY 10029 USA; 3 New York Consortium in Evolutionary Primatology, New York, NY 10016 USA; 4 Vestibular Research Laboratory, School of Psychology, University of Sydney, Sydney, NSW 2006 Australia; 5 Department of Otolaryngology, Icahn School of Medicine at Mount Sinai, New York, NY 10029 USA

**Keywords:** Inner ear, Biological anthropology

## Abstract

Our sense of balance is among the most central of our sensory systems, particularly in the evolution of human positional behavior. The peripheral vestibular system (PVS) comprises the organs responsible for this sense; the semicircular canals (detecting angular acceleration) and otolith organs (utricle and saccule; detecting linear acceleration, vibration, and head tilt). Reconstructing vestibular evolution in the human lineage, however, is problematic. In contrast to considerable study of the canals, relationships between external bone and internal membranous otolith organs (otolith system) remain largely unexplored. This limits our understanding of vestibular functional morphology. This study combines spherical harmonic modeling and landmark-based shape analyses to model the configuration of the human otolith system. Our approach serves two aims: (1) test the hypothesis that bony form covaries with internal membranous anatomy; and (2) create a 3D morphometric model visualizing bony and membranous structure. Results demonstrate significant associations between bony and membranous tissues of the otolith system. These data provide the first evidence that external structure of the human otolith system is directly related to internal anatomy, suggesting a basic biological relationship. Our results visualize this structural relationship, offering new avenues into vestibular biomechanical modeling and assessing the evolution of the human balance system.

## Introduction

Our sense of equilibrioception (balance and 3D spatial orientation) is among the most central of our sensory systems, particularly in the evolution of human positional behavior. This evolutionarily ancient sense is comprised of the vestibular system and is essential for providing a gravitational frame of reference for many biological processes (including postural behavior^1–4^, gaze stabilization, for a review see^5^; and cognitive functions^4^; see also^6,7^. Indeed, the vestibular system is fundamental to how we perceive ourselves within 3D space^8^.

**Figure 1:**
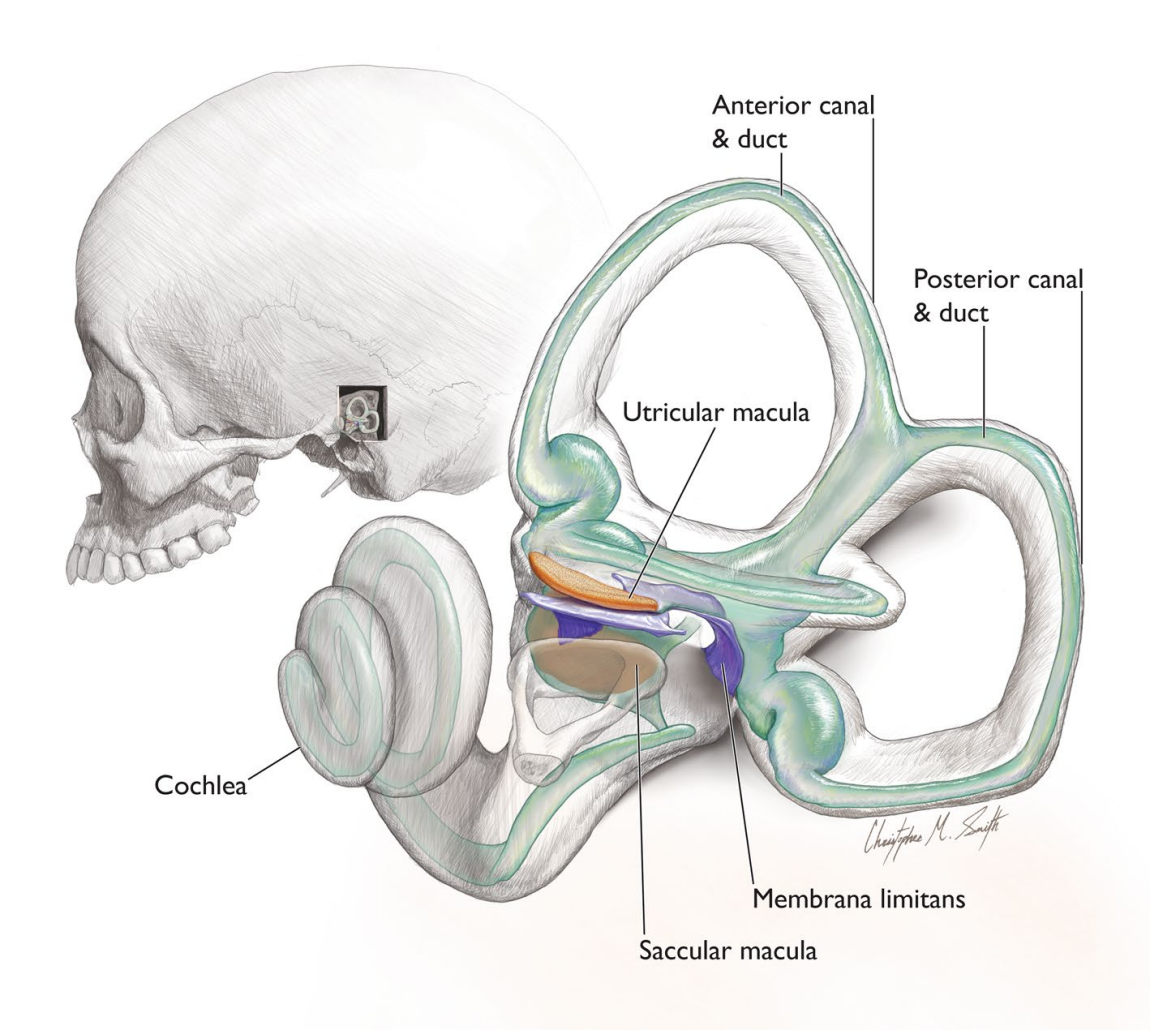
Inner ear anatomy. (**A**) Left lateral view of inner ear within the human cranium; (**B**) left bony labyrinth enlarged. Membranous labyrinth shown in green; maculae shown in orange. The membrana limitans (blue) is a membranous “hammock” that serves to support the utricle and its neural substrate by attaching to the walls of the bony vestibule. Illustration by CMS. Modified from^9^.

In humans, the sensory end-organs responsible for this sense, collectively called the peripheral vestibular system (PVS), are comprised of fluid-filled membranous structures (membranous labyrinth) housed within a bony “shell” (bony labyrinth) located inside the petrous temporal bone alongside the bony and membranous components of the cochlear (peripheral auditory) system. The human PVS includes five paired organs: three semicircular canals (SCCs) with their constituent ducts and two otolith organs (the utricle and the saccule) housed within a bony “entryway” (vestibule). The SCCs detect angular acceleration of the head, while the utricle and saccule detect changes in horizontal and vertical linear acceleration, respectively and therefore also sense vibration and head tilt relative to gravity. The utricle is a sac-like membranous structure to which all three semicircular ducts connect. The utricle and its neural substrate lie upon a thin membrane known as the membrana limitans (ML)^9^. The saccule is a relatively smaller membranous sac lying just inferior to the utricle (see Fig. 1 for an illustration of the anatomy). The otolith organs are surrounded by a perilymphatic space typically referred to as the periotic cistern (or cisterna periotica vestibuli/periotic cistern of Streeter)^10^. The utricle and saccule each have a region of sensory epithelium (macula) along either the inferior or medial wall, respectively. While the saccular macula is attached to bone, the utricular macula is tethered to bone only at its anterior end, with the remaining portion lying upon the ML (along with its neural substrate). These maculae contain sensory hair cells and supporting cells underlying a gelatinous matrix containing calcium carbonate crystals (otoconia). It is the displacement of the otoconia in the maculae during linear acceleration or tilting of the head that causes the hair cells to fire, sending signals to the brain. Due to these functional properties of the otolith organs, morphological differences influence functional properties (e.g., maculae orientation, organ shape, etc.)^11–14^.

The difficulty in examining the PVS, and inner ear more broadly, is due in part to its inaccessibility within the hardest bone of the body, the petrous portion of the temporal bone (derived from the Greek word for “rock”; *petra*). Historically, examination of inner ear structure began with gross dissection and was later advanced with microscopic and histological techniques. Following centuries of research using such methods, x-ray computed tomography revolutionized 3D visualization of inner ear structure through virtual segmentation and reconstructions of form. More recently, high-resolution micro-computed tomography (μCT) and synchrotron-phase contrast imaging have allowed the visualization of previously poorly understood structures of the membranous labyrinth^15–18^. Specifically, the combination of μCT modalities with 3D digital modeling and shape analyses has established the spatial configuration of the ML^9,19^ and morphological structure of the ductus reuniens (DR), a link between the peripheral hearing and balance mechanisms^20^. While advances in visualization technology and shape modeling indeed provide novel insight into inner ear structure, our lack of understanding relationships between internal structure and the external contiguous bony framework has limited investigations into the core of the PVS: the otolith system.

Knowledge of the relationship of bone to underlying soft tissues has been an important part of historical attempts to uncover structural, and by extension, functional relations in regions of the vertebrate skull. From Leonardo’s attempts to uncover the seat of the soul in the senso commune by using measurements to understand location (for reviews see^21,22^) to uncovering the interface between the brain and skull^23^, researchers today continue to push the boundaries of how to visualize the relationships between internal vs. the external^24–26^. Most recently, and directly related to the PVS, are the significant advances in understanding the morphological interface of the bony SCCs and their internal duct system.

The primate SCCs (both external and internal structure) are well visualized and understood. In particular, the bony structure of the canals reflects the radius of curvature and orientation of their internal membranous ducts^27–31^. As a result, the SCCs are often used as a vehicle to study aspects of evolutionary change such as differences in positional behavior^32–35^, and phylogenetic relationships among taxa^36–38^. Furthermore, advances continue to be made to enhance our understanding of the relationships between internal and external SCC morphology using modeling techniques to extract biomechanical properties of the SCC system in fossils (e.g.,^17,39,40^).

**Figure 2:**
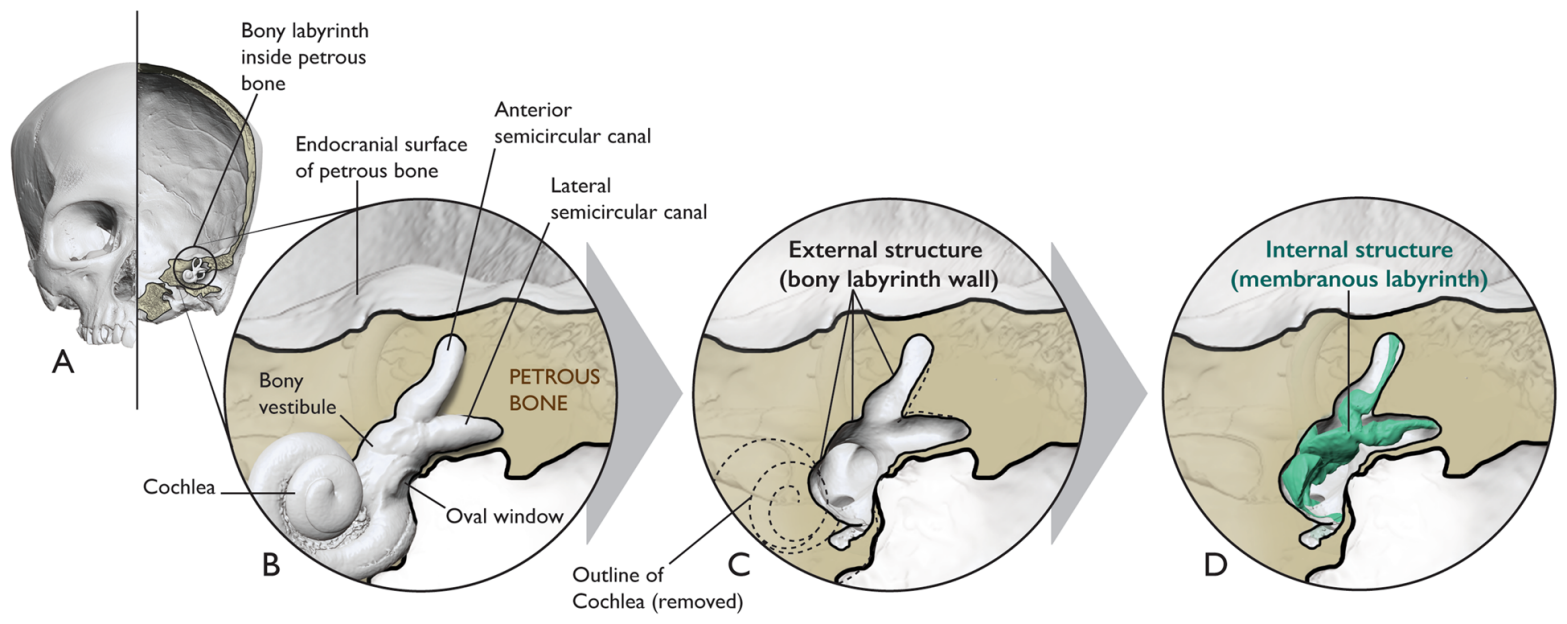
Schematic of the external vs. internal structure of the inner ear. The left bony labyrinth is shown in coronal cross-section of the cranium (**A**) and enlarged in (**B**). The bony labyrinth is depicted here as a 3D model derived from μCT data. We use the term “external structure” to refer to the walls of the bony labyrinth which are part of the petrous bone (**C**). We use the term “internal structure” to refer to the membranous walls of the inner ear sensory end-organs which are encapsulated by the external structure (**D**).

Compared to the SCCs, we know very little about the relationship between internal and external structure of the much less studied otolith system (Fig. 2). This lack of knowledge stems from the inability to examine its seemingly vague and irregular bony structure, hindering our attempts to establish recognizable and reproducible landmarks. Consequently, relationships between bony structure and the internal membranous anatomy of the otolith organs remain unknown, precluding a comprehensive understanding of PVS function and evolution.

This study tests the hypothesis that bony form (size + shape) of the vestibule correlates with the internal membranous anatomy of the otolith organs. Our method involves using osmium tetroxide contrast enhanced micro-computed tomography (μCT) imaging of human temporal bones (n = 13), digital visualizations, spherical harmonic-based shape descriptions (SPHARM), and landmark-based 3D shape analyses of bony and membranous structure. We assess overall correlation between hard and soft tissue using ordinary least squares (OLS) and two-block partial least squares (2B-PLS) regressions. Results indicate a significant association between the hard and soft tissues of the otolith organs (*p* < 0.05) and relative orientation of the maculae to surrounding bony structure (*p* < 0.005). These data support our hypothesis and provide the first evidence that bony structure of the vestibule reflects internal otolith organ anatomy.

We subsequently generate a standardized 3D morphometric model of the otolith system to visualize the spatial configuration of both bony and membranous constituents. We model the 3D morphological structure of the human otolith system by combining SPHARM and landmark-based shape analyses which yields the average shape (SPHARM) and spatial configuration (landmarks) of the bony vestibule, membranous utricle, and membranous saccule. This comprehensive morphometric model thus utilizes landmark and landmark-free components to extract bony otolith organ structure from an otherwise nebulous bony form and pairs it with corresponding soft tissue structure. Our approach elucidates the otolith system’s spatial relationships to other inner ear structures and the cranium. This novel conception of the human otolith system has implications for biomechanical modeling of vestibular function and understanding evolutionary changes to the PVS throughout human evolution.

## Results

**Figure 3:**
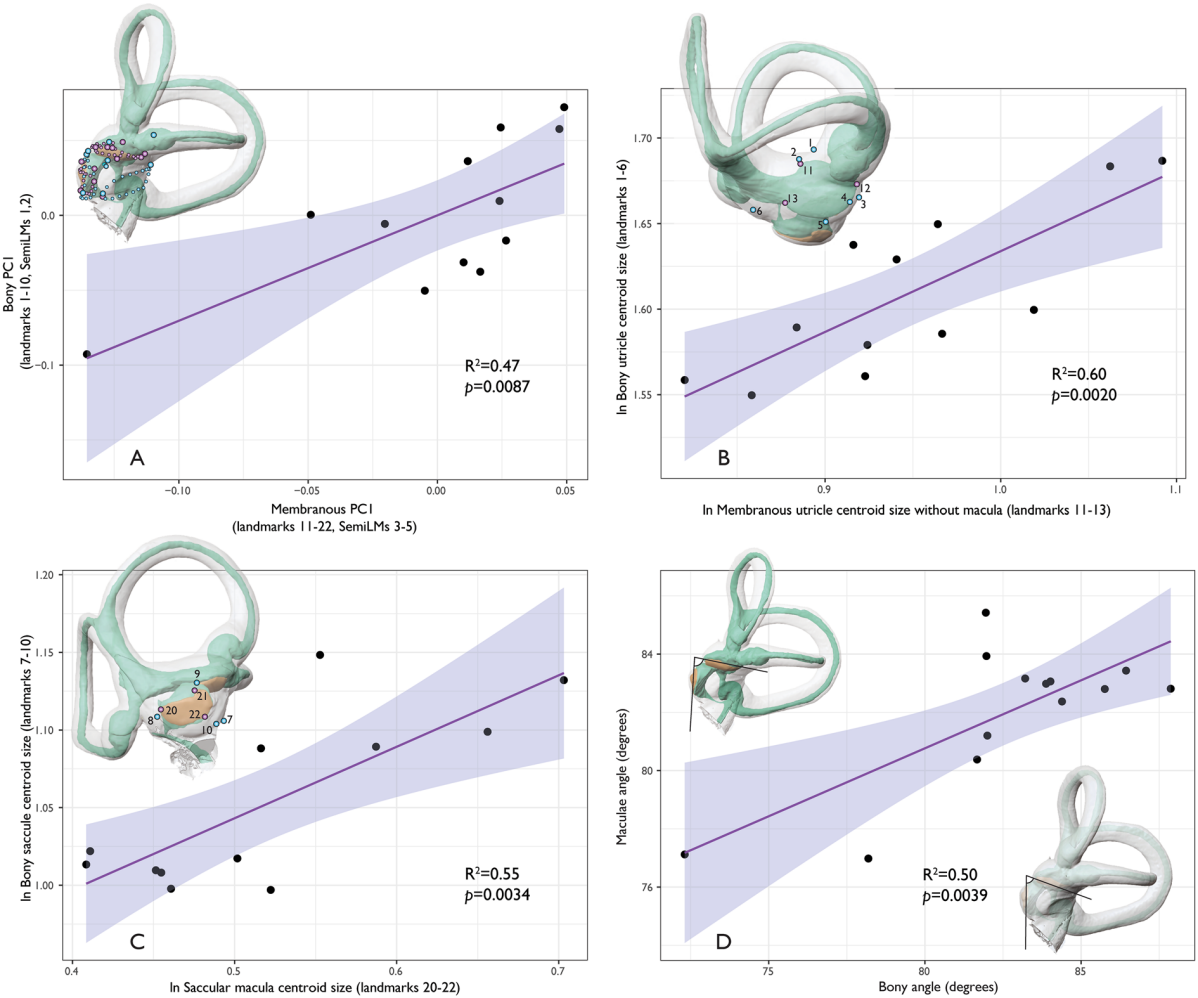
Bivariate plots of best fit OLS regressions of: (**A**) first principal components of the PCAs based on bony and membranous shape variation; (**B**) natural log-transformed centroid size of the bony utricle and membranous utricle; (**C**) natural log-transformed centroid size of the bony saccule and saccular macula; and (**D**) two sets of angles between: (1) planes fit to each macula (maculae angle); and (2) planes fit to a set of three bony landmarks surrounding the utricle (LMs 2, 4, and 6) and saccule (LMs 8–10) (bony angle). There are significant correlations between size and shape of the otolith organs and surrounding bone as well as between macula planes reconstructed from bony landmarks and from the maculae themselves. Purple: membranous landmarks; blue: bony landmarks.

Results indicate a significant correlation in overall shape variation between the bony and membranous tissues of the otolith organs (*p* = 0.009; R^2^ = 0.47), and between the bony and membranous tissue of the utricle (*p* < 0.026; R^2^ = 0.35) and saccule (*p* < 0.002; R^2^ = 0.60) when using both single landmarks and sliding semilandmark curves (see Fig. 3A; Supplementary Table 5). When removing certain landmarks (landmark sets 2–5), the relationship between hard and soft tissue shape variation of the otolith organs becomes non-significant. There are also significant correlations between centroid size of the bony and membranous components of the otolith organs. In particular, centroid size of all landmark arrangements of the bony utricle correlates with utricular membranous and macular landmarks (*p* = 0.04–0.002; R^2^ = 0.29–0.60) (see Fig. 3B; Supplementary Table 6). Size of the utricular macula alone, however, does not correlate with any bony utricular landmark arrangements (Supplementary Table 6). Centroid size of the saccular macula significantly correlates with both bony saccule landmark arrangements (*p* = 0.01–0.003; R^2^ = 0.41–0.55) (Fig. 3C; Supplementary Table 6). Centroid size of membranous saccule and macula landmarks are only correlated with landmarks on the spherical recess (Supplementary Table 6).

Angles between approximated and actual maculae orientation were the lowest when using landmarks 2, 4, and 6 to estimate the plane derived from landmarks 17–19 on the utricular macula (Mean = 4.62° ± 1.75°, range = 1.26°–7.52°; Supplementary Fig. 6; see Supplementary Table 7 for a description of the angles used to estimate bony plane approximation of maculae). In every case, the plane of the utricular macula is tilted superiorly relative to the bony angle in the lateral aspect. Differences for estimating saccular macula orientation were lowest between landmarks 8–10 and a selection of the spherical recess (Mean = 4.58°  ± 2.67°, range = 1.32°–9.95°; Supplementary Fig. 6) or to landmarks 20–22 (Mean = 4.53° ± 2.20°, range = 1.23°–7.59°). In every case, the plane of the saccular macula is tilted laterally relative to the bony angle in the posterior aspect.

Relative orientation of planes fit to otolith organ maculae (landmarks 17–19 and 20–22) significantly correlates with planes fit to surrounding bony structure (landmarks 2,4,6 and 8–10) (*p* < 0.005; R^2^ = 0.50) (Fig. 3D). The point at which the posterior bony ampulla meets the vestibular aqueduct (we term this the aqueductal point) is particularly important to approximate the orientation of the utricular macula. This is reflected in a better fit of the approximated-to-actual utricular macula plane when the aqueductal point is included (see Supplementary Fig. 6). Many of the above bony landmarks derive from bony attachments of the ML (see Supplementary Tables 3, 4, and 7 for descriptions and justifications of landmarks and bony planes used to approximate maculae).

**Figure 4:**
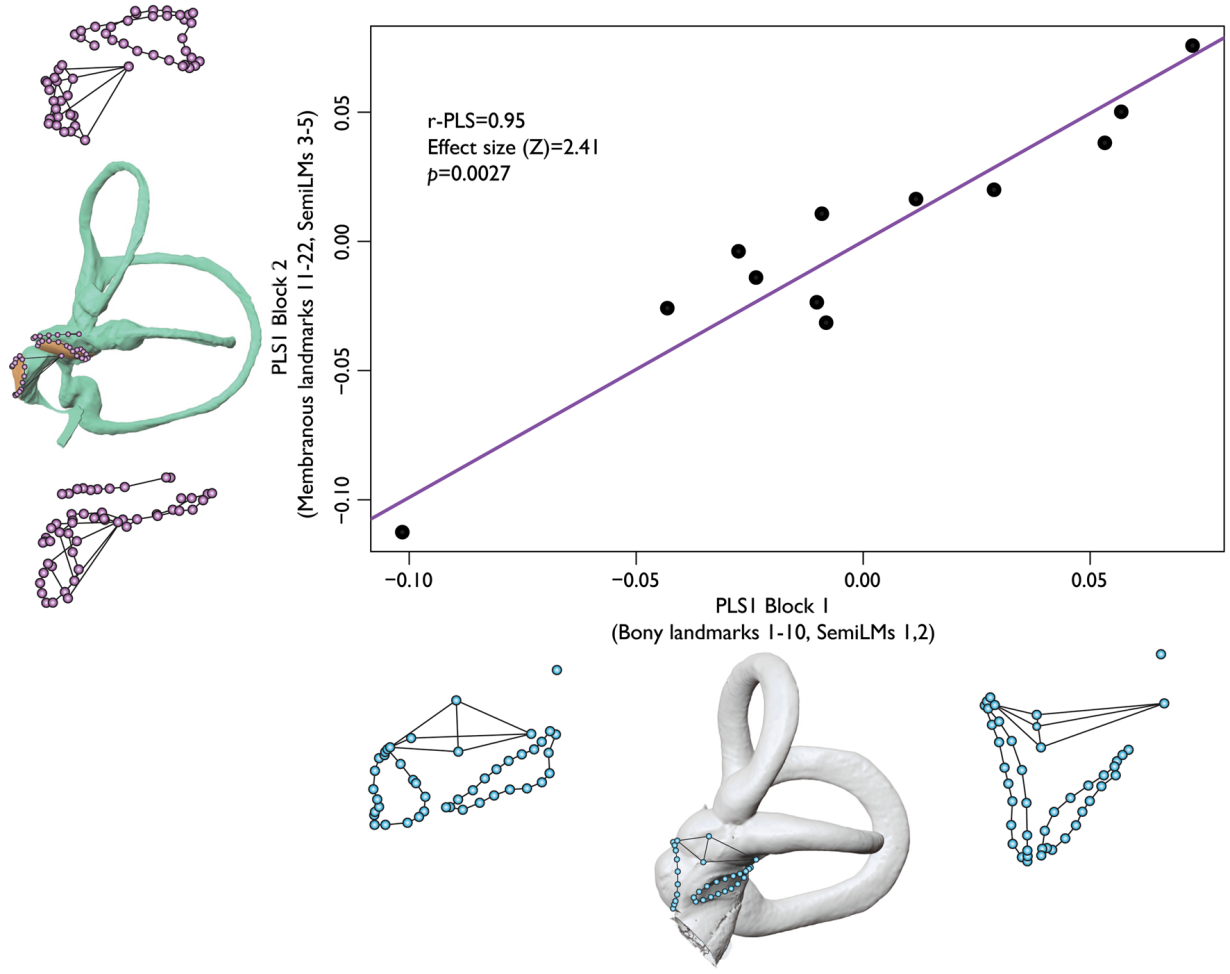
Integration plot from the 2B-PLS analysis. Data show significant correlation of overall shape variation between Procrustes superimposed landmark coordinates on bony and membranous otolith organ structure. Wireframes show extremes of shape variation for each block that covaries with the other block. The x-axis shows the extent of shape variation in bony landmarks and sliding semilandmark curves (shown in blue). The y-axis shows the extent of shape variation in membranous landmarks and sliding semilandmark curves (shown in purple). Center images of the x and y axes depict mean landmark configurations superimposed on the bony or membranous labyrinth, respectively.

Our analyses also show significant levels of integration and modularity among all landmarks (Fig. 4). The degree of modularity, however, lessens and becomes non-significant when considering single landmarks only (Supplementary Table 8). This is likely due to the numerous points included in the sliding semilandmark curves capturing more subtle shape variation within each module. This makes it appear that there is more variation within, rather than between modules. Overall, subset 3 shows significant integration, but reduced modularity, indicating that this bony landmark configuration captures overall internal shape variation of the otolith system. While bony and membranous tissue of the utricle show significant integration with and without semilandmarks, integration within the saccule is driven by a strong association between the saccular macula and spherical recess (see Supplementary Table 8). Caution should be exercised, however, when interpreting single summary statistics (e.g., *p*values) of geometric data based on biological structure due to the inherent multivariate nature of landmarking analyses on living forms^41^. Such statistics are gross oversimplifications but provide a starting point to explore features of biological size and shape.

Removal of sliding semilandmark curves and various landmarks (landmark subsets 2–5) has a negligible effect on the parameters of the integration tests. When the semilandmarks around the oval window are removed from the analysis, overall integration becomes non-significant (*p* = 0.129), suggesting that the oval window is structurally related to the morphology of the human otolith system. Levels of integration between the utricle and saccule are also significant, along with significant integration between hard and soft tissue when the utricle and saccule are considered independently from one another.

### The morphometric model of the human otolith system

The second aim of this paper is to provide a visualization of the human otolith system, both detailed and schematized to communicate a recognizable bony structure that can be applied to study evolutionary change of this system in humans. Combining mean meshes derived from our SPHARM modeling with consensus landmark arrangements, we can now model the morphology and spatial configuration of the otolith system for the first time. The membranous utricle has a flattened, “pancake-like” morphology that is oblong with a mediolaterally expanded and superoinferiorly compressed anterior region. This anterior region contains the utricular macula, which spans from the posterior bound of the supraovalic fossa to the utricular crest anteriorly and crista vestibuli medially. Posterior to this, the utricle is mediolaterally compressed and begins to increase in height superoinferiorly as it merges into the base of the common crus, lateral semicircular duct (non-ampullated side), and the posterior semicircular duct.

**Figure 5:**
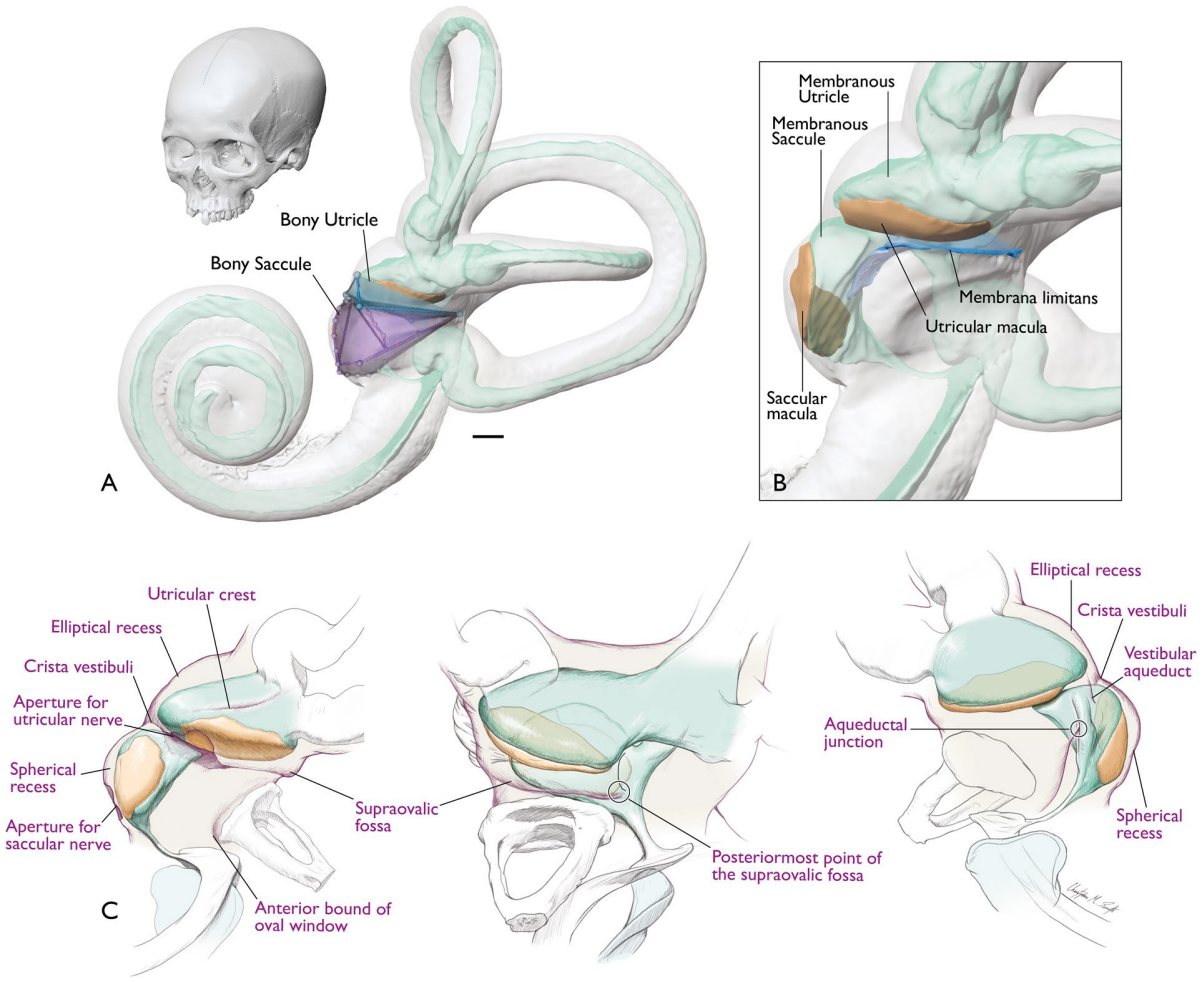
Anatomy of the human otolith system. (**A**) Schematized model of bony utricle and bony saccule structure (blue and purple wireframes, respectively) in anterolateral view superimposed onto the membranous labyrinth and ML. Cranium shows head orientation. (**B**) Enlarged view from A showing membranous structure. (**C**) Drawing of the labyrinth (purple) outlining bony features that reflect membranous otolith organ structure (green) and maculae (orange). Scale bar = 1 mm. Illustration by CMS.

**Figure 6:**
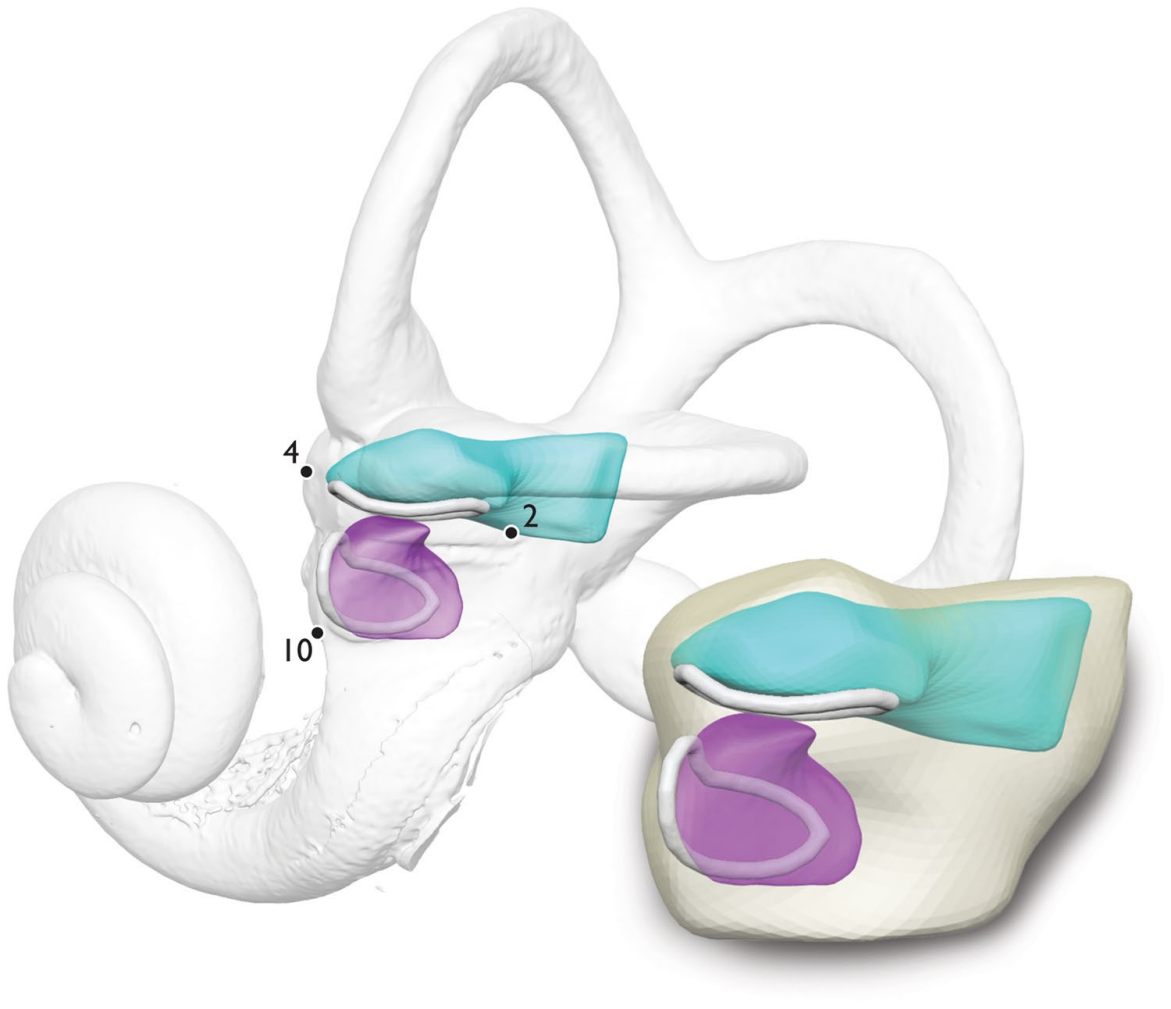
The morphometric model of the human otolith system. SPHARM-PDMs depict the mean bony vestibule (transparent light yellow), mean utricle (teal) and mean saccule (purple). SPHARM-PDMs are aligned using the consensus landmark configuration. Maculae contours (representing wireframes and shown here in white) are based on consensus semilandmark configurations. Numbers indicate important bony landmarks for estimating maculae configuration (for landmark descriptions see Supplementary Table 3). For a turntable video of this model see Supplementary Video 1.

The membranous utricle contacts two structures: (1) the anterior bony wall of the vestibule whereby the utricular nerve meets the upward curve of the utricular macula; and (2) the ML which runs along its entire inferior border. The remainder of the membranous utricle contacts perilymph and its overall contour is reflected in the shape of the encapsulating walls of the bony vestibule that directly surround it (see Figs. 5 and 6, and Supplementary Video 1). This bony housing of the utricle (bony utricle) is bounded laterally by the supraovalic fossa, medially by the elliptical recess, posteriorly by the aqueductal point, and inferiorly by the ML spanning from the supraovalic fossa to the crista vestibuli. The inferior aspect of the membranous utricle and utricular macula are positioned at the level of the crista vestibuli and supraovalic fossa (see Fig. 5 for visualizations of these descriptions).

The membranous saccule has a curved, sac-like shape which contacts the medial bony wall of the vestibule where the saccular nerve meets the saccular macula. The entirety of the saccular macula contacts bone as it follows the contour of the spherical recess. The superior portion of the membranous saccule projects laterally and contacts the ML at its superior margin just inferior to the utricular macula. The remainder of the membranous saccule contacts only perilymph. The bony housing of the saccule (bony saccule) is bounded posteriorly by the crista reuniens, superiorly by the crista vestibuli, medially and inferiorly by the spherical recess, and laterally by the oval window (see Figs. 5 and 6, and Supplementary Video 1). The spherical recess houses a significant portion of the membranous saccule.

By using thin-plate spline warping of the morphometric model to a particular configuration of bony landmarks (i.e., the landmark arrangement on the original specimen) we can estimate the morphology and positioning of the membranous utricle and membranous saccule in relation to the bony vestibule. When testing the effectiveness of this approach, our method recovers significantly lower differences in shape (distances between vertices in meshes) compared to distances among actual specimens (see Supplementary Fig. 8). The mean distance between estimated and original vertices is 0.05 mm (± 0.16 mm), while the maximum distance recovered is 0.72 mm and interquartile range is 0.17 mm (Supplementary Fig. 8). Visualizing estimated vs. original mesh distances as heatmaps show the largest mesh distances in the superolateral region of the membranous utricle (see Supplementary Fig. 9). Reconstructions of the saccule more closely approximate their original morphology and configuration (most vertices are within 0.2 mm of original mesh).

We present our morphometric model in two parts. First, Fig. 5 shows a detailed illustration of the anatomy. Second, the average SPHARM-PDM derived mesh is shown in Fig. 6 aligned to the consensus landmark configuration and superimposed onto the bony labyrinth of one individual from our sample (also see Supplementary Video 1 for a 3D representation of the model).

## Discussion

Our results indicate a significant relationship between the external structure of the human inner ear otolith system and its internal anatomy, supporting our initial hypothesis. This suggests a basic biological relationship between bone and otolith organs. Such a relationship likely reflects a broader pattern of integration within inner ear as the otic epithelium of the developing membranous labyrinth does indeed mediate chondrogenesis of the otic capsule^42^.

Deciphering these relationships now enable a more standardized description of the human otolith system in the form of a morphometric model that encompasses hard and soft tissue. The average SPHARM-PDM models provide a starting “template” from which to reconstruct the shape and size of the membranous otolith organs placed into position based on a given bony landmark configuration. These findings offer new avenues to investigate the function and evolution of the human vestibular system by: (1) contributing to enhanced biomechanical vestibular modeling and (2) establishing bony features of the human otolith system to enable the reconstruction of internal membranous anatomy from bone.

### Morphometric modeling of the human otolith system

Our morphometric model visualizes the structure of the otolith system by utilizing SPHARM-PDM modeling to establish average morphology conformed to a consensus landmark-based configuration (Fig. 6). The SPHARM-PDM model establishes shape while the landmark analyses create the framework upon which to translate, rotate and scale this morphology. The overall shape of the human otolith system can be characterized as a bony “shell” (vestibule) taking on a twisted triangular shape convexly curved in the medial direction. This bony vestibule is spanned anteroposteriorly by a membranous “hammock” called the membrana limitans (for a review see^9^). The membrana limitans divides the vestibule into a superior part (housing the membranous utricle) and an inferior part (housing the membranous saccule), both containing perilymphatic fluid.

The bony attachments of the ML form the basis for most bony landmarks included in this study. Our results confirm that these indeed reflect otolith organ structure. Such bony features include the crista vestibuli, supraovalic fossa, utricular crest, the border of the spherical recess, and aqueductal point. Several landmarks placed on these bony features track variation of the underlying otolith organs, in both overall form and orientation of the maculae (landmarks 1–10, see Supplementary Table 3). More specifically, our bony landmarks approximate maculae orientation within 4.5 and 4.6 degrees (on average) for the utricular macula and saccular macula, respectively. Our results suggest that these bony landmarks are useful in assessing otolith organ morphology when only bony structure is available.

Including the aqueductal point in reconstructed maculae planes improves fit to the maculae planes. This suggests a tight relationship between maculae structure and positioning of the vestibular aqueduct. We hypothesize this is due to the attachment of the ML to this location. Since the ML supports the utricular macula, this may therefore link the aqueductal point to the orientation of the utricular macula. Indeed, the aqueductal point is an important landmark to be considered in future morphological analyses of the vestibular system. Discrepancies between the reconstructed and actual orientation of the maculae could also be affected by distortion of the soft tissues during fixation and/or staining. Further testing of these relationships will add vital data to these modeled relationships.

### Vestibular modeling

Our results have important implications for biomechanical modeling of the otolith system. In particular, by characterizing the average shape of the bony and membranous walls of the otolith system and the positioning of the membranes relative to bone, new data can be obtained on vestibular membrane dynamics. While the walls of the membranous labyrinth are often modelled as rigid^44–46^, more recent biomechanical models indicate the importance of membrane dynamics in the ear. For example, thickly-walled utriculo-canal morphology^47,48^ and supporting trabecular meshwork in the perilymphatic space^49^ contribute to reduced stress proclivities in the mammalian pars superior. In addition, we now know more about tendencies for membrane displacement in the pars inferior, particularly in cases of differential fluid pressures^49–51^. These morphological changes to the membranous labyrinth, along with others such as those acquired during aging^52^ or vestibular endolymphatic hydrops^53^ can alter flow dynamics (as observed in models of the utricle^44^) leading to differential vestibular transduction. Indeed, a more thorough understanding of the membranous labyrinth in relation to perilymphatic structure and encasing bony anatomy would aid in further unifying otolith organ macromechanics. Our model provides a way to describe and explore these macromechanics in a 3D morphometric framework.

Our morphometric model also presents a new approach to reconstruct 3D soft tissue of the inner ear using bony data. This modelling approach has the capability to reconstruct the morphology and positioning of the otolith organs relative to the bony vestibule within an average of 0.05 mm compared to the original mesh (see “Results”). Most differences encountered between the modeled and actual meshes relate to the superolateral region of the membranous utricle (see Supplementary Fig. 9). These differences may relate to a more plastic shape of the superior utricle as the inferior wall and macula of the utricle are more stabilized by the ML (see Fig. 5B). In contrast to the superolateral utricle, the morphology and positioning of the entire membranous saccule is well estimated with most of the reconstructed vertices within 0.2 mm of the original mesh (see Supplementary Fig. 9). Combining 3D landmarking with SPHARM methods enables new ways to reconstruct soft tissue structure. Such approaches could provide alternative ways to model biomechanical properties of the membranous labyrinth in a given individual when only bony data is present.

### Evolutionary implications

Bony morphology of the inner ear provides a means to assess various aspects of vertebrate evolution. In particular, such morphology has been utilized to examine the evolution of positional behavior^32^, auditory capabilities^54^, and physiological processes such as endothermy^39^. Bony labyrinth morphology is also used to reconstruct phylogeny due to its early ossification during prenatal development^55,56^ and its location within the dense petrosal region of the temporal bone. These features render the bony labyrinth resistant to morphological plasticity and taphonomic distortion, respectively, making it a valuable resource for assessing phylogenetic relationships.

In contrast to the usefulness of SCC and cochlear morphology, the otolith system has long been neglected in evolutionary studies of the inner ear. This derives from the idea that bony morphology of the vestibule does not reflect the internal membranous morphology of the otolith organs. Our results refute this hypothesis. Bony structure of the human vestibule indeed relates to internal anatomy. The features described above reflect the internal morphology of the otolith system through the lens of bony structure. Our approach enables exploration of evolutionary change in the human otolith system by modeling morphological relationships of the membranous utricle and membranous saccule to bone and creates reproducible bony landmarks that reflect internal membranous structure. In turn, these new data can be used to assess morphological change in skeletal samples and ultimately reconstruct vestibular soft tissue, providing a basis for testing biomechanical models of vestibular function.

One particular aspect of functional morphology that this model can assess is maculae orientation as approximated through bony landmarks. Maculae orientation is vital to the functional properties of the otolith system. Specifically, orientation of the macula relative to the temporal bone and gravity vector is known to influence the directional coding of each macula^12–14,57^. While there is a great deal of interindividual variability in the morphology of the utricular macula^12^ and saccular macula^58^, our model provides a novel set of bony landmarks that reliably approximates the orientation of the maculae using the following bony features: the supraovalic fossa, utricular crest, aqueductal point, and border of the spherical recess. These bony features can contribute to examinations of maculae orientation in skeletal specimens. In turn, such studies could shed light onto differential directional coding of these organs. Such measures would be especially useful when combined with functional metrics of the SCCs and ducts (e.g., canal size and cross-sectional diameter of duct lumen) to give a comprehensive picture of vestibular evolution.

Our data provide the first evidence that external structure of the human inner ear otolith system is directly related to internal anatomy enabling the creation of a visual morphometric model. This standardized description establishes new avenues into vestibular biomechanical modeling (e.g., membrane dynamics) and the assessment of evolutionary change at the core of the vestibular labyrinth. Indeed, the otolith system’s central role in many physiological processes highlight the importance of better understanding its functional morphology. When combined with other vestibular data (e.g., SCC metrics), this model can enhance our understanding of spatial relationships between the otolith organs and cranial structure, providing the foundation to assess the evolution of a prime sensory system alongside significant anatomical changes throughout human evolution.

## Materials and methods

### Data acquisition

Our sample comprises thirteen human otic capsules from eleven adult individuals (Supplementary Table 1). Provenance and sex for this sample is unknown. μCT of temporal bones stained with osmium tetroxide shows, at high resolution, both bony and membranous structures was therefore chosen as our imaging modality^19,59^.

This study does not include any living human participants. The temporal bone scans are from archival cadaveric cranial specimens stored at the Royal Prince Alfred Hospital. All procedures on human temporal bones were approved by the NSW Department of Health. Procedures were approved by the Royal Prince Alfred Hospital Ethics Office (protocol number X19-0480) and experiments were performed in accordance with relevant guidelines and regulations.

Each temporal bone was drilled to isolate the otic capsule and was fixed for 24 h in Karnovsky’s fixative (3% paraformaldehyde, 0.5% glutaraldehyde in phosphate buffer). This process has been documented to minimize shrinkage^60^. They were then soaked in 2% osmium tetroxide, for up to 9 days, and scanned in a 64-bit μCT scanner (MicroXCT-400; Xradia, Pleasanton, California, USA) with voxel resolution ranging from 8.3 μm (0.0083 mm) to 25 μm (0.025 mm). Average scan time was 8 h. The μCT data in tagged image file format (.TIFF) files were cropped to focus on the vestibule. Details on methods, including scanning parameters, have been described in detail in earlier publications^18,19^.

The stained μCT stack of .TIFF images were imported into the software 3D Slicer, version 4.10.1^61^. This process enabled 3D reconstruction of the following structures: bony labyrinth, membranous labyrinth, utricular macula, and saccular macula. Each bony labyrinth was segmented using semi-automated thresholding to define the boundary between the fluid-filled space of the labyrinth and surrounding bone. Each macula and membranous labyrinth were manually segmented. Segmentations were exported as .OBJ virtual meshes and imported into the software Zbrush, version 2021.6.6^62^ where the surface topology was cropped (if needed), surface smoothed, and artifacts removed in surface meshes.

### Data analysis

Our data analysis and modeling process comprise two modalities:

1. Spherical harmonic analysis (SPHARM) to mathematically describe the form of the human otolith system at its simplest levels.
2. Landmark-based assessment of hard and soft tissue 3D form (size + shape) comprised of four analyses: ordinary least-squares (OLS) linear regressions of principal components of shape, centroid size, and maculae orientation, and tests of modularity and integration.

Combination of the above two modalities creates a comprehensive morphometric model which visualizes relationships among bony and membranous features of the otolith system. We subsequently test the accuracy of the model in estimating membranous otolith organ morphology from bony landmarks. Any part of this model (e.g., particular sets of landmarks) can be used to investigate aspects of structure and their relationships to other parts of the inner ear and cranium.

#### Spherical harmonic analysis (SPHARM)

To objectively model the fundamental shape of the bony vestibule and otolith organs, we created a series of Point Distributed Models using Spherical Harmonic representation (SPHARM-PDMs) of membranous utricles and membranous saccules from a sample of five otic capsules from five individuals (for descriptions of SPHARM principles see^63,64^). These five otic capsules were used as they provide the only complete sets of utricular and saccular membranes. Spherical Harmonic analysis lends itself to analysis of the vestibule due to its roughly spherical shape (excluding the SCCs). We also created SPHARM-PDMs of ten bony vestibules which include otic capsules from the above membranous sample. In order to generate the SPHARM-PDMs, we first created cropped meshes of the vestibule and otolith organs (see protocol for cropping in Supplementary Fig. 1 and Supplementary Table 2). This was done by remeshing each mesh in Meshlab, version 2020.07^65^ using quadric edge collapse decimation (number of faces/vertices was set to 30,000/15,000 for bony vestibule respectively, 10,000/5,000 for membranous utricle and 5,000/2,500 for the membranous saccule). The decimated meshes were then smoothed using the Taubin smooth function (settings as default—λ = 0.5; µ = −0.53; 10 smoothing steps). Then we Boolean subtracted the maculae from the membranous otolith organs, creating an “imprint” of these sensory regions in the membranous wall. We Procrustes-aligned all virtual meshes derived from μCT segmentations (see “Data acquisition” section above) using the “Auto 3dGM” plugin in 3D Slicer^66,67^. This shape-alignment utilizes a two-phase process which aligns subsamples of pseudolandmark sets placed on each mesh and once again with a higher number of pseudolandmarks. For our analysis we set the number of points for Phase 1 to 50 and Phase 2 to 200. To create binary labelmaps for the SPHARM-PDM spherical analysis we transformed the aligned Surface Model meshes into binary labelmaps and exported the volumes for the bony vestibule, membranous utricle, and membranous saccule.

Cropped meshes do not include what some term the “posterior utricle”^17^ or “inferior utricle”^68,69^. We view this region concomitant with and functionally related to the posterior duct and ampulla due to its constricted diameter and perpendicular orientation to the plane of the membranous utricle. This likely alters endolymphatic fluid dynamics from those present in the membranous utricle itself^44,70,71^.

SPHARM-PDMs were created in 3D Slicer using the SlicerSALT project (https://www.salt.slicer.org^72^). To ensure accurate topology correspondence of the meshes, volumes were rescaled with X, Y, Z spacing parameters set to the default 0.05 mm for the membranous utricle and membranous saccule and 0.01 mm for the bony vestibule (in order to capture more subtle bony detail). Subdivision level for the output meshes was set to 15. All other settings were set as default. Models were created at SPHARM degrees 1, 2, 3, 4, 5, 10, 15, and 25 to capture the most fundamental components of shape (Supplementary Fig. 2). Models did not exceed SPHARM degree 25 as shape becomes obscured by topological noise at higher degrees.

#### Landmark-based assessment of hard-soft tissue 3D shape covariation

A set of landmarks and sliding semilandmark curves was designed to capture the morphology of the bony vestibule and internal soft tissue (for a total of 22 single landmarks and 86 semilandmarks; see Supplementary Figs. 3–5 and Supplementary Table 3). This was done in order to test our hypothesis of correlation within the vestibule. The approach comprises four analyses examining relationships between bone and membranous structure. These include OLS linear regressions of principal components of shape (analysis 1), centroid size (analysis 2), and maculae orientation (analysis 3), and tests of modularity and integration (analysis 4). Landmark selections for analyses 1 and 4 were carried out in six successive sets (see below) to determine an arrangement of bony landmarks that best reflect the internal membranous structure of the otolith organs. Twelve of the original thirteen specimens were used for landmarking since specimen #7 lacked adequate soft tissue preservation to properly landmark. Landmarking was conducted in 3D Slicer by CMS. The composite structure comprised of these landmarks we term the “otolith system” as it captures structural variation of not only the membranous otolith organs but of surrounding periotic and bony structure. (see Supplementary Table 4 for justification of landmark selection).

Landmark sets used for analyses 1 and 4 (Supplementary Figs. 3–5):

1. We chose an initial set of 22 landmarks (Type I and Type II) and five sliding semilandmark curves to analyze covariation between hard and soft tissue of the otolith system (see Supplementary Table 3; for a review on landmark types and usage see^73–75^). Bony landmark selection was based upon our prior research on the relationships among otolith organ, ML, and bony vestibule morphology^9,19,76^. Membranous landmarks were chosen to reflect internal structure of the otolith system^43,77,78^.
  We chose a landmarking approach for these analyses of covariation instead of pseudolandmarking^67^ or landmark-free methods^79,80^ to reduce the number of extraneous points and avoid issues related to landmark homology (for a review see^81,82^). Using such methods may conceal observable, reproducible, and structurally important landmarks for bony areas that covary with membranous anatomy. Our chosen bony landmarks also avoid the foramina of the utricular and saccular nerves (known as the macula cribrosa) which perforate the capsular wall to innervate the maculae, causing irregularities in segmentation of the bony labyrinth.
2. This landmark set is the same as above, but the sliding semilandmark curve around the oval window (SemiLM2) has been removed. This set was chosen to determine the level of association the oval window has with internal membranous structure.
3. This set removes all sliding semilandmark curves, reducing the number of landmarks to only include single landmark points. This set was chosen to track covariation more precisely within the otolith system related to internal membranous structure.
4. This set uses a reduced number of landmarks from set three (landmarks 1–4, 6–10 for bony landmarks, and 11–22 for membranous landmarks), removing landmark 5 since it is shown to have the highest intraobserver variance in placement (see below).
5. This set uses landmarks from set 4 but removes landmark 7. Removal of landmark 7 at the anteriormost point of the oval window determined if this point is important for tracking variation in internal membranous structure.
6. A final collection of sets divides the landmarks into those of the greater utricle and greater saccule (“greater” indicating the bony and membranous components of the utricle and/or saccule). Sets include bony landmarks 1–6, and membranous landmarks 11–13, 17–19, and SemiLMs 3 and 4 for the greater utricle and bony landmarks 7–10 and membranous landmarks 14–16, 20–22, and SemiLMs 1, 2, and 5 for the greater saccule. This was done to determine the relationship of bone to soft tissue within these respective regions.

### Analysis 1: principal shape components linear regressions

For each landmark set above, coordinates underwent Generalized Procrustes superimposition using the “gpagen” function in the “geomorph” package (version 4.04) in the software R, version 3.5.3 (83). For those analyses including sliding semilandmark curves, these points were first resampled for equidistant placement along the trajectory of their respective curve. It is possible that equidistant placement can lead to statistical artifacts and may not represent geometric or biological correspondence among specimens^84^. Therefore, during Generalized Procrustes superimposition, semilandmarks were allowed to slide along the tangent directions of their curves while minimizing bending energy in order to optimize the homology of landmark positions^84^. It should be noted, however, that sliding of semilandmarks cannot match true biological homology^85^.

Generalized Procrustes superimposition was carried out before rather than after the division of landmarks into bony and membranous subsets. This is often termed the “within a configuration” approach and places all landmarks into a common shape space preserving relative size and positioning of landmark subsets^85–88^. This approach was chosen to retain information about how the subsets are connected to one another since the membranous and bony labyrinths are connected via the perilymph, trabecular meshwork of connective tissue, and the ML^89,90^.

Procrustes-aligned landmark coordinates were then divided into the bony and membranous portions for each of the six landmark sets above. Each bony and membranous landmark set was then subject to a principal component analysis (PCA) using the “gm.prcomp” function in the “geomorph” package of R to assess overall shape variation and determine the component of shape that accounts for the largest amount of shape variation (PC1). A series of eleven OLS models were created using the primary membranous shape component (membranous PC1) as the explanatory variable and primary bony shape component (bony PC1) as the response variable (Supplementary Table 5). This was done using the “lm” function of the “stats” package in R.

### Analysis 2: centroid size linear regressions

A subset of single landmarks from Set 3 above was used to evaluate the relationship between size of the bony and membranous structure of the otolith organs. We were specifically interested in the sizes of each membranous otolith organ compared to the bony structure directly surrounding it (e.g., bony utricle vs. membranous utricle). We, therefore, did not use centroid sizes from the above six landmark sets, but instead used centroid sizes from five bony and four membranous subsets of single landmarks to simplify interpretation of size relationships (see definitions in Supplementary Table 6 for descriptions of landmark subsets). A series of 15 OLS models were created using the log-transformed centroid size from one of the four membranous landmark subsets as the explanatory variable and the log-transformed centroid size from one of the five bony landmark subsets as the response variable (Supplementary Table 6). This was done using the “lm” function of the “stats” package in R.

### Analysis 3: maculae orientation

Our third analysis examined correlations between measurements of maculae orientation using landmarks on the bony vestibule and the maculae themselves. Historically, maculae orientation and configuration within the head has been measured using tests of otolithic reflexes^91^, wax-plate reconstructions^92^, wax-plate reconstructions projected within a stereotaxic system^93^, and computer-aided reconstruction based on serially sectioned temporal bones^94,95^. Here we measure ipsilateral maculae orientation to one another using the angle formed between planes of best fit created from three landmarks either on bone or the maculae.

We first tested the accuracy of how well bony plane angles match angles derived from macular planes. Eight sets of angle comparisons were tested for the utricular and saccular maculae (see Supplementary Fig. 6 and Supplementary Table 7). Bony planes approximating the utricular macula were constructed using either landmarks 2, 4, and 5, or 2, 4, and 6. Landmarks 2 and 6 were chosen because they represent consistent attachment points of the ML which supports the utricular macula^9^ and landmark 4 was chosen as it matches the superiormost boundary of the anterior curve of the utricular macula. Landmark 5 was included as it approximates the inferomedial bound of the elliptical recess. Bony planes approximating the saccular macula were constructed using either a selection of the spherical recess or landmarks 8–10. Landmarks 8–10 were chosen as they give the orientation of the spherical recess, a bony fossa within which sits the saccular macula^76^. For a review of the bony landmarks see Supplementary Tables 3 and 4.

Maculae planes were reconstructed using two methods: (1) selecting the entire macular surface on a 3D mesh derived from μCT data; and (2) three landmarks on each macula^17–19,20–22^ (see Supplementary Table 3). All planes were created, and measurements taken using the software Geomagic Wrap 2017^96^. The set of bony planes that best matched each macula (see Supplementary Fig. 6) where then regressed against maculae planes of best fit using OLS regression in R to determine if the bony planes significantly correlate with maculae planes.

### Analysis 4: tests of modularity and integration

We carried out tests of integration using 2B-PLS analyses (based on 10,000 random permutations) and tests of modularity on all six landmarks sets above and compared levels of association among them to determine the set of bony landmarks that best relate to internal structure of the otolith system. These tests were carried out using the “integration.test” and “modularity.test” functions in the “geomorph” package in the software R^83^. The “gmShiny” graphical interface (version 0.1.3) was also used for analysis and visualization of results^97^.

The 2B PLS method and RV coefficient, more specifically, are sensitive to sample size (n) and number of variables^98^. Therefore, due to our small sample sizes, we instead use effect size (z) and p-values to determine strength of integration and covariance ratio (CR) to determine degree of modularity among landmark subsets.

#### Measurement error and landmark variance

We tested intraobserver error by landmarking a single BL (with respective maculae) ten times, reorienting the mesh to a random initial orientation before landmarking. The largest Procrustes distance recovered was 0.025, which is considerably less than the smallest Procrustes distance between individuals (0.136) and within one individual (0.07). Therefore, intraobserver error does not have a substantial effect on landmark placement.

We also conducted an analysis of bony landmark coordinate variance in 3D Slicer to determine landmarks that vary substantially compared to others (semilandmarks were excluded). Of all landmarks, landmark 5 (the point at which the elliptical recess meets the spherical recess in vestibular horizontal view) is the most variable.

### Morphometric model

The combination of SPHARM and landmarking modalities enables the creation of a morphometric model which establishes relationships among hard and soft tissue of the otolith system. To create the model, we first determined the mean shape of the SPHARM-PDM for the bony vestibule, utricle and saccule in SlicerSalt (based on the 25° bony vestibule and saccule SPHARM-PDM, and 15° utricle). The degree level of the utricle was chosen to be lower than for the bony vestibule due to its oblong shape which hinders SPHARM’s ability to map it to a parametrization sphere at higher degree levels.

We then aligned the mean SPHARM meshes of the bony vestibule, utricle, and saccule to the position of the landmark consensus model (single landmarks only; Set 3) using Fiducial registration-similarity transform in 3D Slicer (translation, rotation, and scale; Fig. 6; process of alignment shown in Supplementary Fig. 7). This combination of mean SPHARM meshes and consensus landmarks gives the orientation, position, shape, and size of the membranous utricle and membranous saccule relative to the bony vestibule. Consensus semilandmark configurations were then used to construct average contour wireframes of the utricular macula (SemiLM4) and saccular macula (SemiLM5).

In order to test the efficacy of our morphometric model in reconstructing soft tissue structure we replicated the membranous otolith organs of each specimen used in the SPHARM-PDM analysis (n = 5, see Supplementary Table 1) using only bony landmarks and subsequently compared the new estimated mesh to the original. To accomplish this, the three aligned meshes (membranous utricle, membranous saccule, and bony vestibule) of the average morphometric model were transformed into binary labelmaps in 3D Slicer. The membranous utricle and membranous saccule were then subtracted from the labelmap of the bony vestibule, resulting in a final binary labelmap of the “negative space” between the otolith organs and bony vestibule. This space represents what would normally be filled with perilymphatic fluid in life. We transformed this final labelmap into a surface mesh we call the “average perilymphatic mesh”.

The average perilymphatic mesh was then landmarked with bony landmarks 1–10 in 3D Slicer and warped to the bony landmark coordinates of each individual using the “warpRefMesh” function in the “geomorph” package of R. This function uses thin-plate spline morphing to warp a given 3D mesh to a set of landmark coordinates. The resulting warped perilymphatic meshes were then imported into the software Zbrush where the surface topology of the bony vestibule was removed, leaving only the modeled membranous utricle and membranous saccule. The modeled membranous utricle and membranous saccule were exported from Zbrush as a single .PLY mesh. Distances between vertices of the modeled .PLY meshes and the original meshes of the membranous utricle and membranous saccule were calculated and visualized using the “meshDist” function in the “Morpho” package (version 2.10) of R. Wilcoxon rank sum tests (with continuity correction) were used to determine if there was a significant difference between: (1) distances of the modelled meshes to the original specimen; and (2) distances among the five original specimens. Before analysis, the original specimens were all aligned to one individual (specimen #9, see Supplementary Table 1) using the surface registration module (similarity transformation) in the “SlicerCMF” plugin for 3D slicer.

## Data availibility

All data generated and analyzed for this study are included in this manuscript and the supplementary information files.

## Supplementary Information

Supplementary Information 1.

Supplementary Information 2.

Supplementary Information 3.

Supplementary Information 4.

Supplementary Information 5.

Supplementary Information 6.

Supplementary Information 7.

Supplementary Video 1.
