# Supplementary material for: First evidence of the link between internal and external structure of the human inner ear otolith system using 3D morphometric modeling
Source: Sci Rep. 2023 Mar 24;13:4840. doi: 10.1038/s41598-023-31235-1 (PMC10039035; doi:10.1038/s41598-023-31235-1)
Supplement: Supplementary file 1 — Supplementary Information 1. [file 41598_2023_31235_MOESM1_ESM.docx]

Supplementary files:

*Please note that the landmarks listed in the R scripts are in a different order than those presented in the manuscript (see supplementary table 9 below). The placement of sliding semilandmark curves were carried out successively, preceded by all bony landmarks and single membranous saccular landmarks and followed by all single membranous utricular landmarks. This order was chosen for ease of placement when manipulating multiple overlapping 3D meshes. The change of landmark order in the manuscript was chosen to improve visualization and communication of results.

The contents of the file folders are as follows:

- Datasets for R scripts:
  - Raw landmarks
  - Sliding semilandmark curves
  - Curve matrices
  - Partition lists for integration and modularity tests
  - Average perilymphatic mesh
  - Landmark file containing landmarks for all 12 specimens and the average SPHARM model (labeled “Model_test.tps”).
- Raw 3D meshes for specimen 9:
  - Bony labyrinth
  - Cropped bony vestibule
  - Membranous utricle and membranous saccule
  - Maculae
- Individual SPHARM-PDM meshes (bony vestibule, membranous utricle, and membranous saccule) for each specimen
- SPHARM morphometric model (aligned to Procrustes consensus landmark coords)
- R scripts:
  - Centroid size linear regressions
  - Otolith system integration and modularity tests
  - Principal shape components linear regressions
  - 3D warping for model test

| **Supplementary Table 9. Raw landmark order and relationships to landmark number from the main manuscript**  Yellow=Bony vestibule; Purple=Membranous saccule; Teal=Membranous Utricle | | |
| --- | --- | --- |
| Raw landmark | Landmark from main manuscript | Landmark description |
| 1 | 1 | Posteriormost point of where lateral bony ampulla meets bony vestibule |
| 2 | 2 | Superior edge of the posteriormost point on the inferior crista of the supraovalic fossa |
| 3 | 3 | Inferior edge of where utricular nerve enters the vestibule (placed along line drawn vertically from anterior bound of the oval window in AVH) |
| 4 | 4 | Inferior edge of superiormost point of utricular crest (placed along line drawn vertically from anterior bound of the oval window in AVH) |
| 5 | 5 | Point at which the elliptical recess meets the spherical recess along the crista vestibuli in AVH |
| 6 | 6 | Inferiormost point where posterior bony ampulla meets the vestibular aqueduct (we term this the aqueductal point) |
| 7 | 7 | Anteriormost point of oval window |
| 8 | 8 | Posteriormost point of spherical recess |
| 9 | 9 | Superiomost point of spherical recess in MVH |
| 10 | 10 | Anteroinferiormost point of spherical recess in MVH |
| 11 | 20 | Posteriormost point of saccular macula |
| 12 | 21 | Posteriormost point of dorsal flap |
| 13 | 22 | Anteroinferiormost point of saccular macula |
| 14 | 14 | Tip of superolateral projection of saccule |
| 15 | 15 | Anterosuperior junction between the saccule and saccular duct |
| 16 | 16 | Anteroinferior junction between saccule and ductus reuniens |
| 17-35 | SemiLM1 | Boundary of where the spherical recess meets the rest of the bony vestibule (19 semilandmarks, resampled, starting at landmark 8, moving superiorly) |
| 36-54 | SemiLM2 | Boundary of the oval window (19 semilandmarks, resampled, starting at landmark 7, moving superiorly) |
| 55-64 | ­­SemiLM3 | Resampled curve spanning from landmarks 12 to 13 (10 semilandmarks) |
| 65-83 | SemiLM4 | Resampled curve along contour of utricular macular surface (19 semilandmarks starting at landmark 17, moving anterolaterally) |
| 84-102 | SemiLM5 | Resampled curve along contour of saccular macular surface (19 semilandmarks starting at landmark 20, moving inferiorly) |
| 103 | 11 | Posteriormost point of utricular hook inferior edge |
| 104 | 12 | Point where utricular ridge meets medial boundary of anterior ampulla |
| 105 | 13 | Point where utricular ridge meets membranous labyrinth posteriorly |
| 106 | 17 | Posteriormost point of utricular macula |
| 107 | 18 | Anteriormost point of the superior edge of curved anterior section of the utricular macula |
| 108 | 19 | Medialmost point of the superior edge of curved anterior section of the utricular macula |
