# Supplementary material for: First evidence of the link between internal and external structure of the human inner ear otolith system using 3D morphometric modeling
Source: Sci Rep. 2023 Mar 24;13:4840. doi: 10.1038/s41598-023-31235-1 (PMC10039035; doi:10.1038/s41598-023-31235-1)
Supplement: Supplementary file 7 — Supplementary Information 7. [file 41598_2023_31235_MOESM7_ESM.docx]

**Supplementary Information**


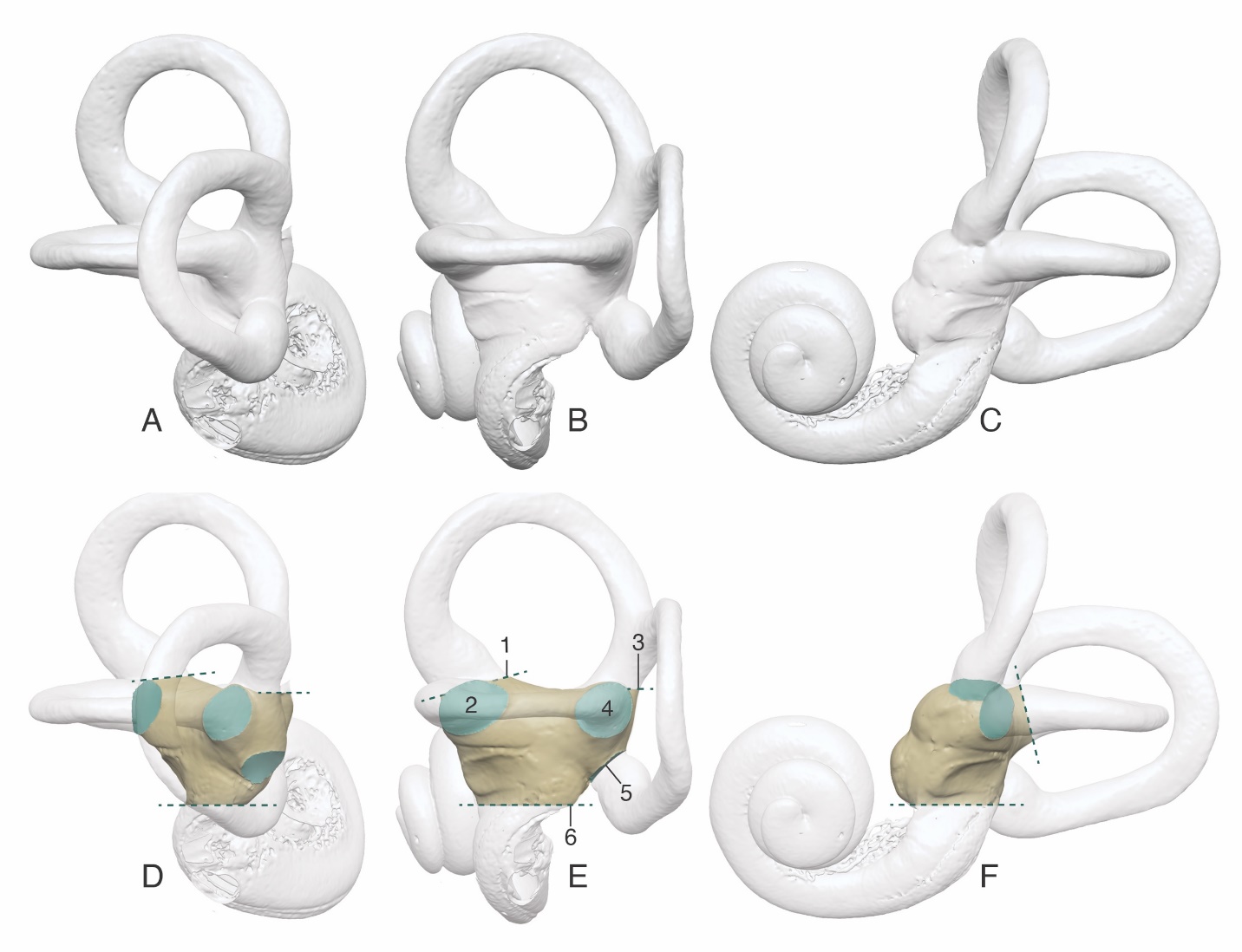


Supplementary Figure 1. Left bony labyrinth of the human inner ear. A) Posterior view; B) Posterolateral view; C) Anterolateral view. D-F show same views as above but include planes upon which the bony vestibule was cropped for the SPHARM analyses. After cropping, each surface was closed using a plane. Numbers correspond to cut planes described in Supplementary Table 2.


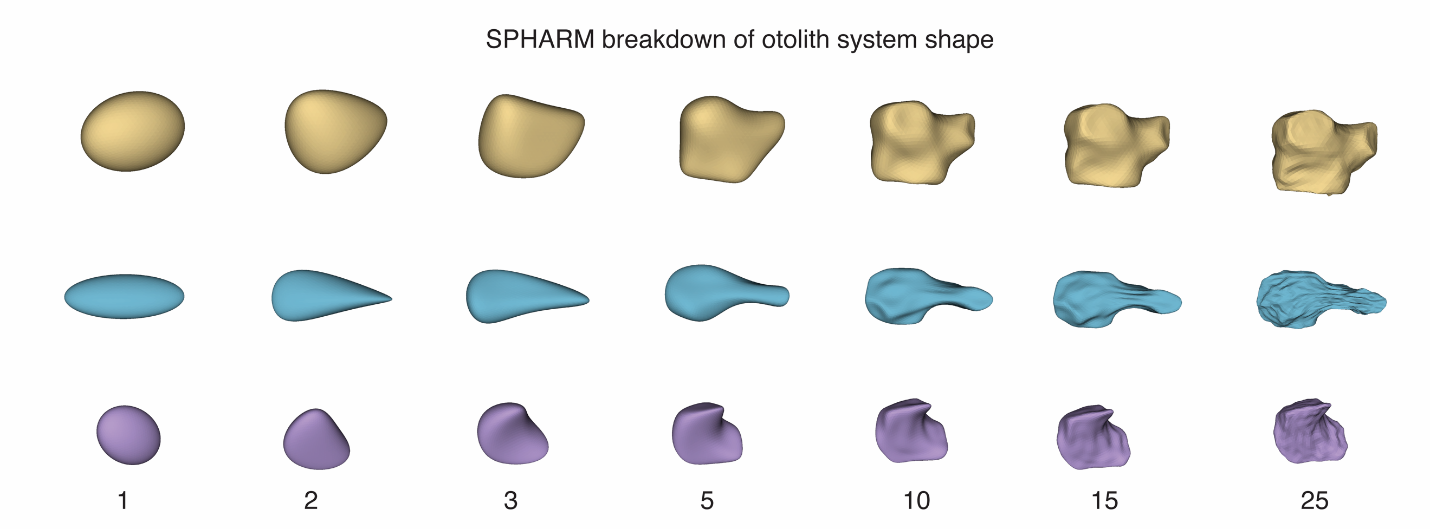


Supplementary Figure 2. Degrees of SPHARM-PDMs from one individual representing the bony vestibule (yellow), membranous utricle (blue), and membranous saccule (purple).


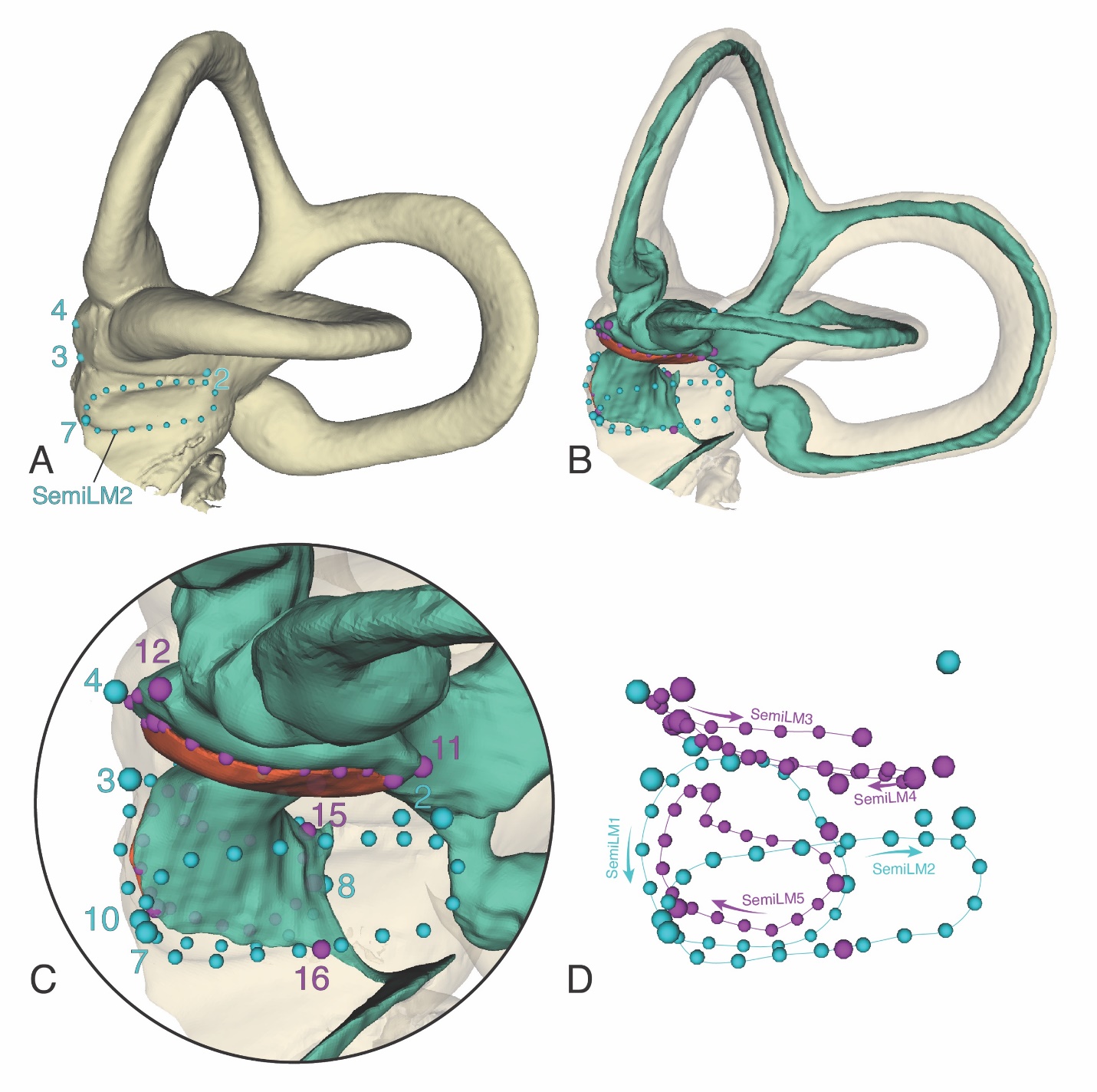


Supplementary Figure 3. Anterolateral view of landmarks and sliding semilandmark curves used in this study. A) Bony landmarks; B) Membranous labyrinth showing both external bony and internal membranous landmarks; C) Close-up of ‘B’ labeling single landmarks; D) Landmarks with semilandmark curves. Arrows show direction of placement for semilandmarks. Bone=Yellow; Membranes=Teal; Maculae=Orange. Bony landmarks=Blue; Membranous landmarks=Purple.


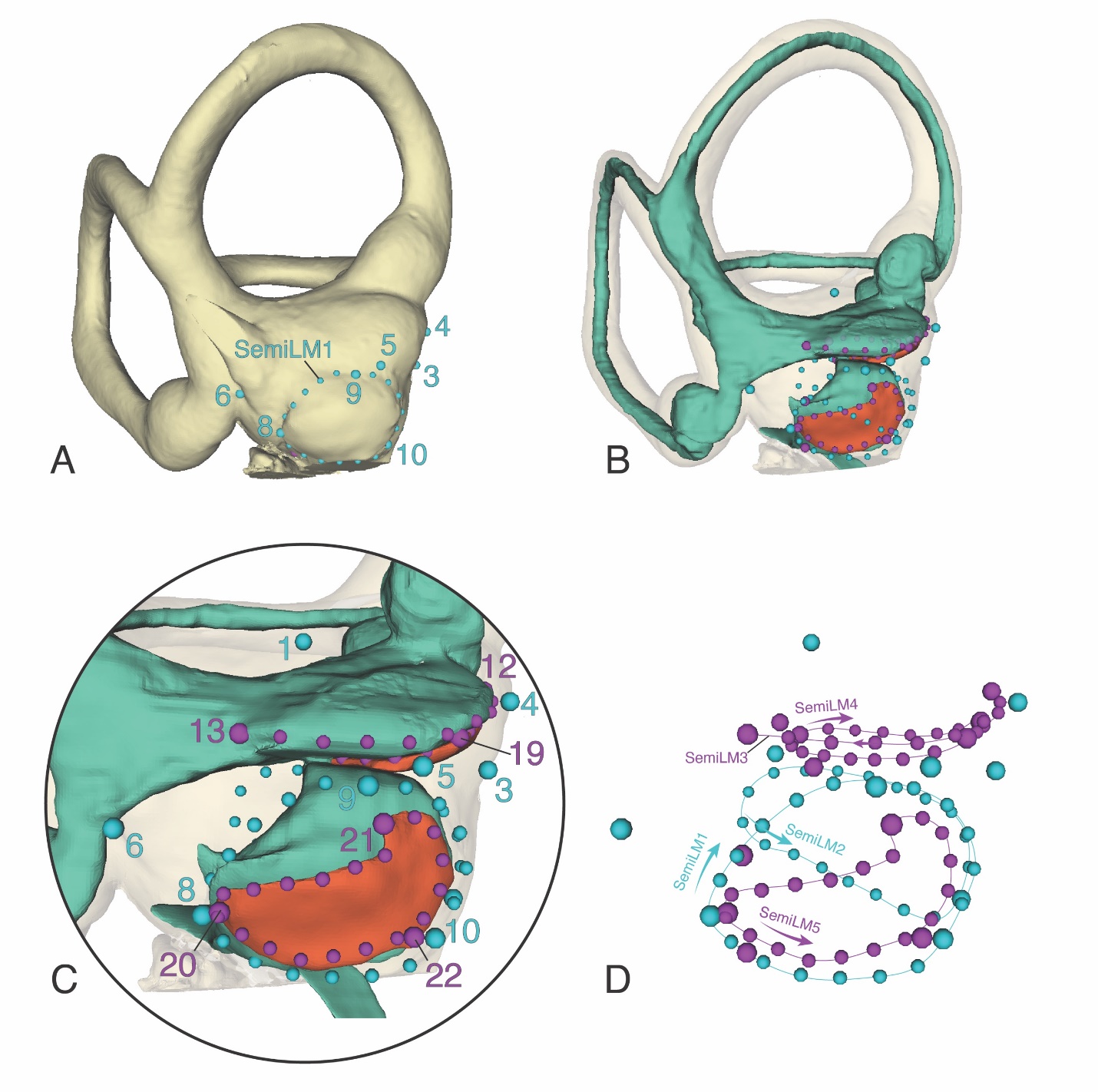


Supplementary Figure 4. Medial view of landmarks and sliding semilandmark curves used in this study. A) Bony landmarks; B) Membranous labyrinth showing both external bony and internal membranous landmarks; C) Close-up of ‘B’ labeling single landmarks; D) Landmarks with semilandmark curves. Arrows show direction of placement for semilandmarks. Bone=Yellow; Membranes=Teal; Maculae=Orange. Bony landmarks=Blue; Membranous landmarks=Purple.


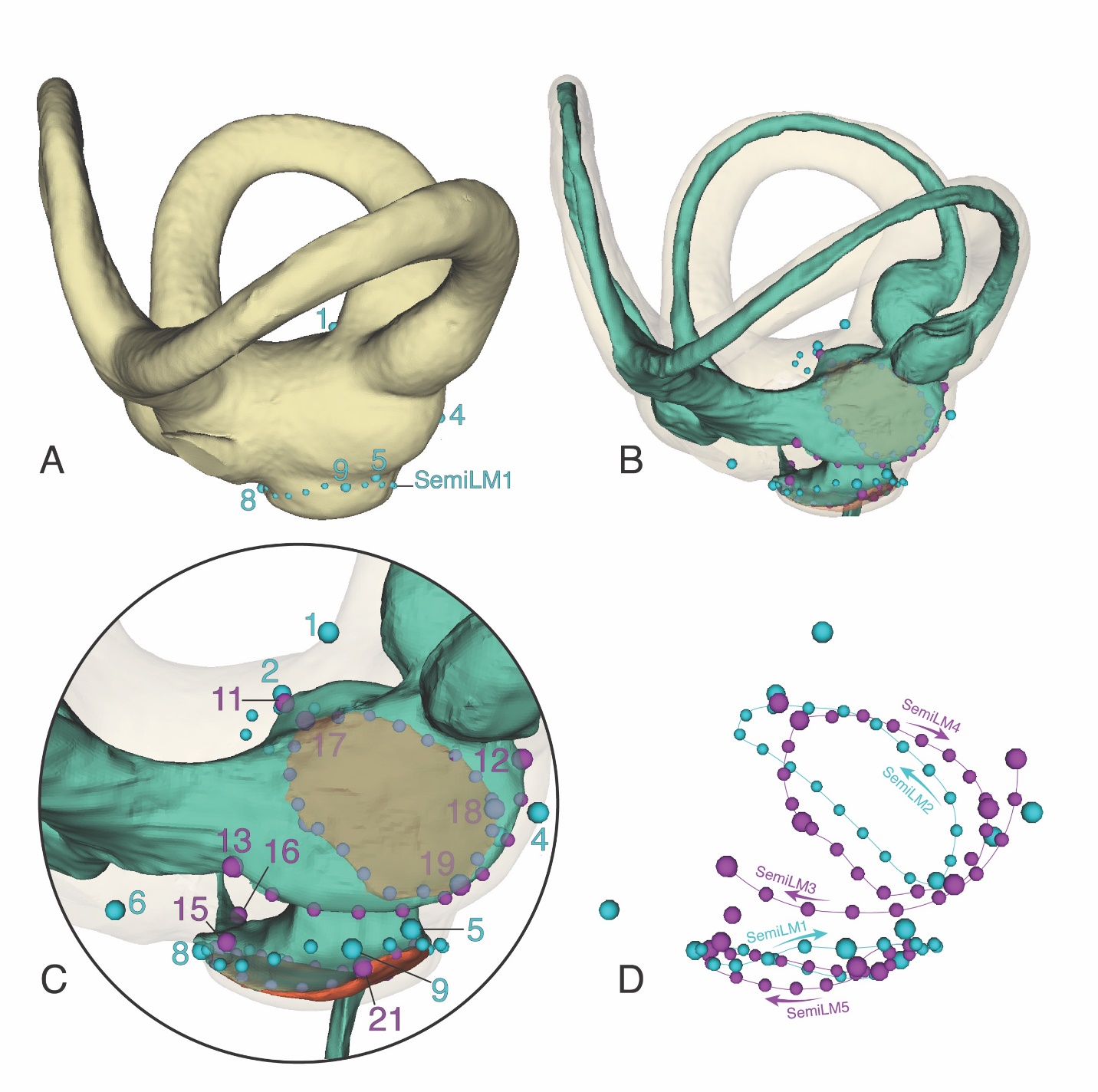


Supplementary Figure 5. Superior view of landmarks and sliding semilandmark curves used in this study. A) Bony landmarks; B) Membranous labyrinth showing both external bony and internal membranous landmarks; C) Close-up of ‘B’ labeling single landmarks; D) Landmarks with semilandmark curves. Arrows show direction of placement for semilandmarks. Bone=Yellow; Membranes=Teal; Maculae=Orange. Bony landmarks=Blue; Membranous landmarks=Purple.


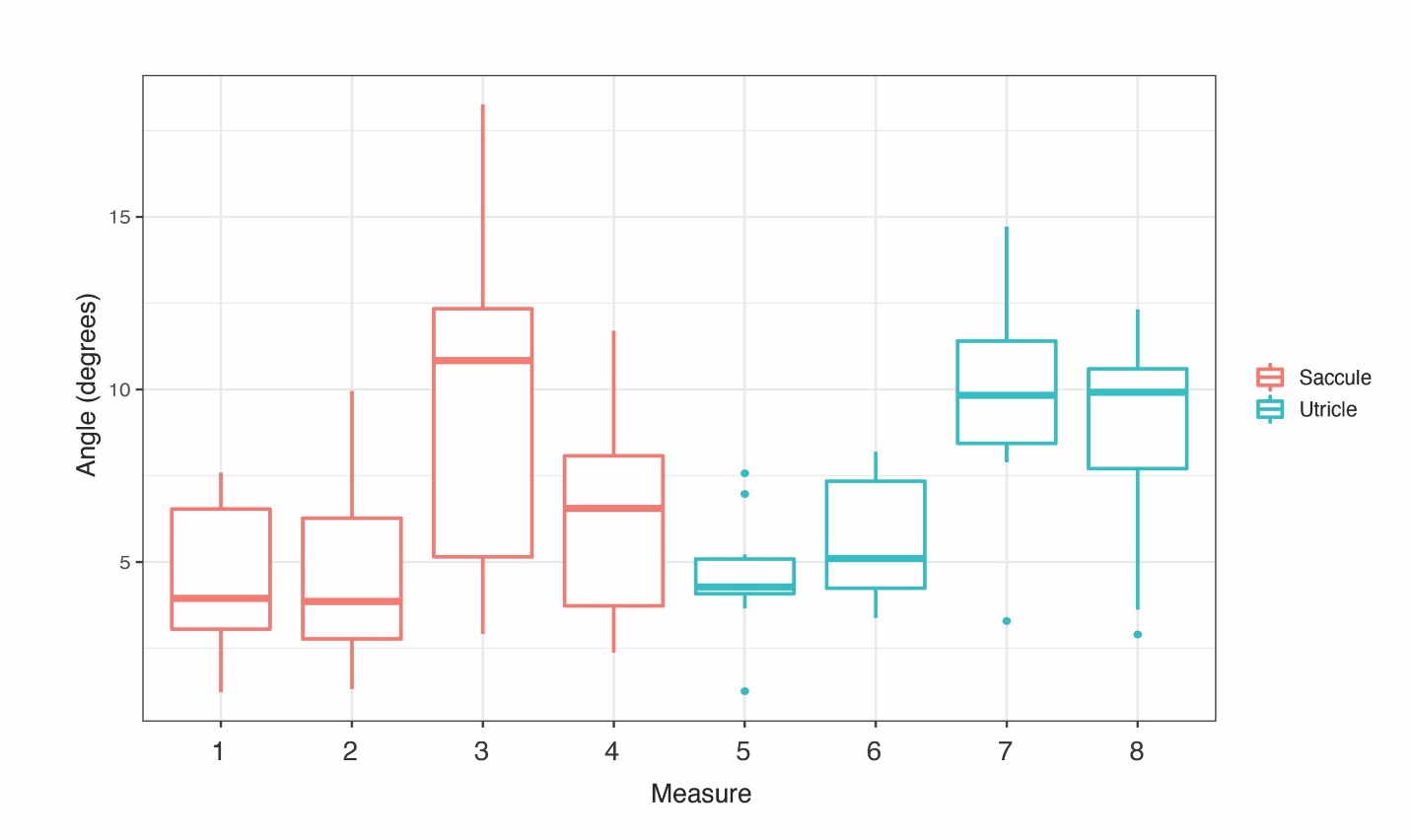


Supplementary Figure 6. Angles (in degrees) between approximated and actual maculae orientation. Landmarks and sets of angles are described in Supplementary Tables 3, 4, and 7.


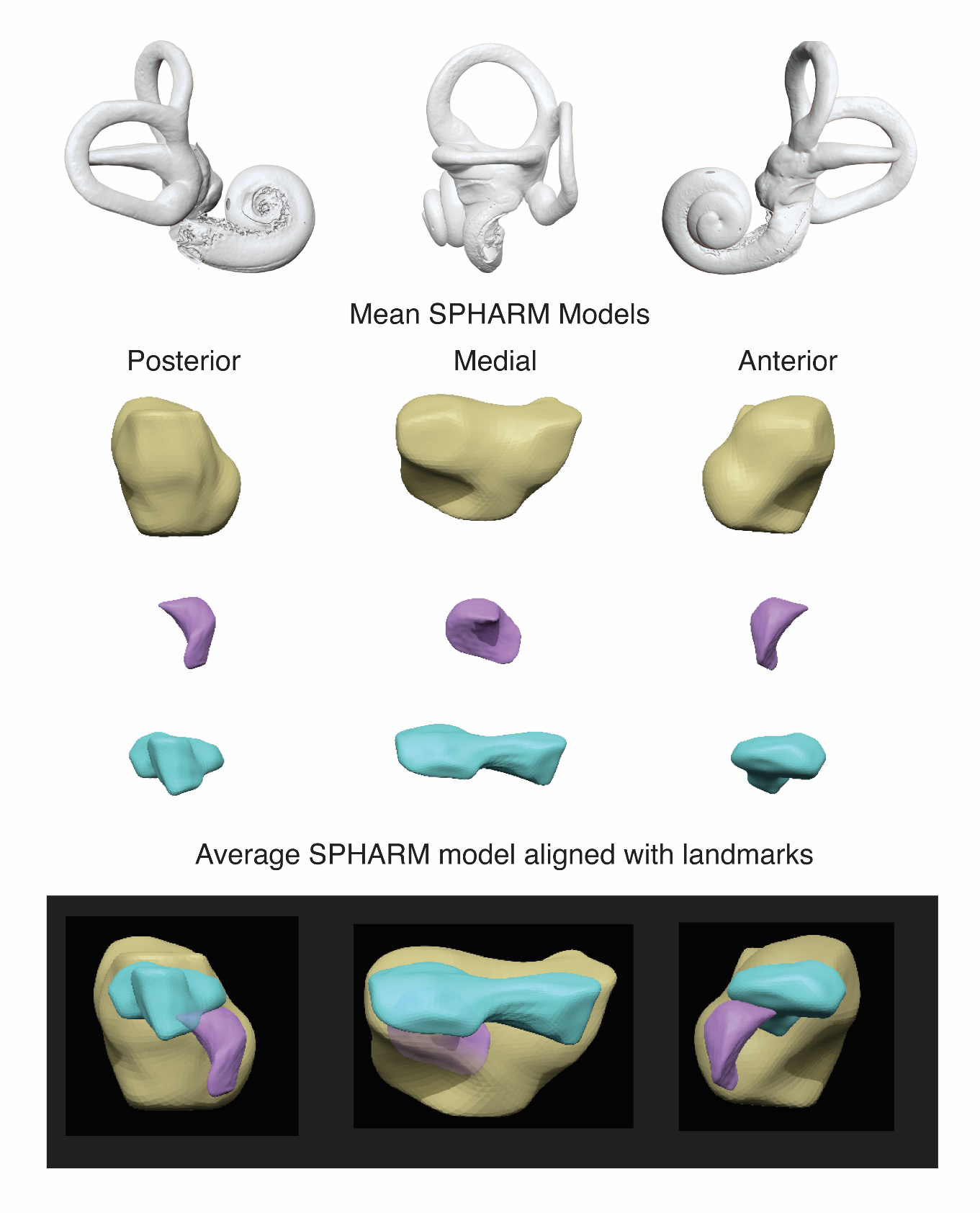


Supplementary Figure 7. Mean SPHARM-PDM meshes. Bony vestibule (yellow), membranous utricle (blue), and membranous saccule (purple) are shown individually above, and aligned using the consensus landmark positions from analysis 1 (shown at bottom).


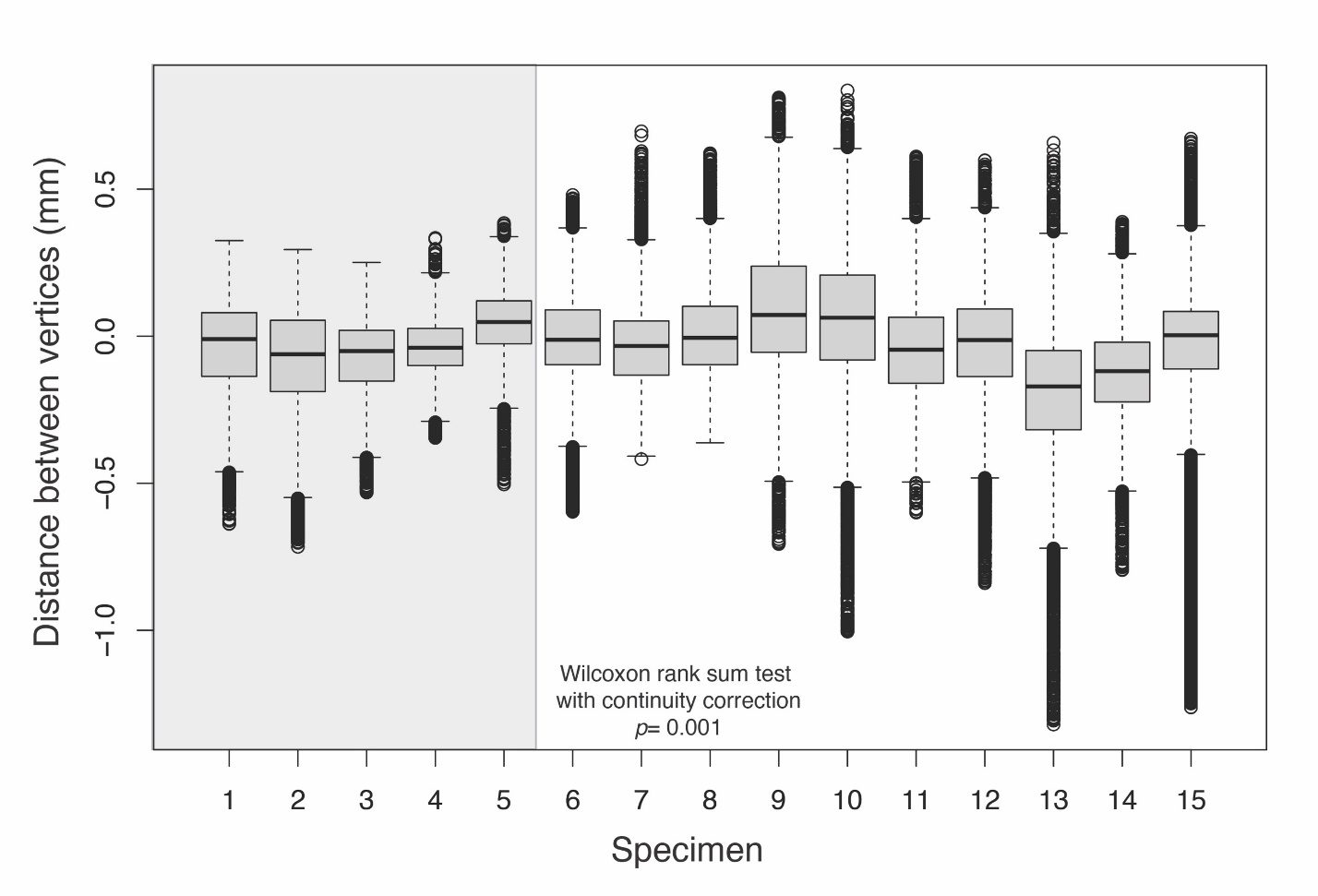


Supplementary Figure 8. Distances between vertices of meshes in mm. Distances between modelled otolith organs and original specimens shown in grayed-out area on left. Distances of vertices among original specimens only shown on right. There is a significant difference between modelled and original distances with those between modelled and original specimens being closer to zero (*p*=0.001).


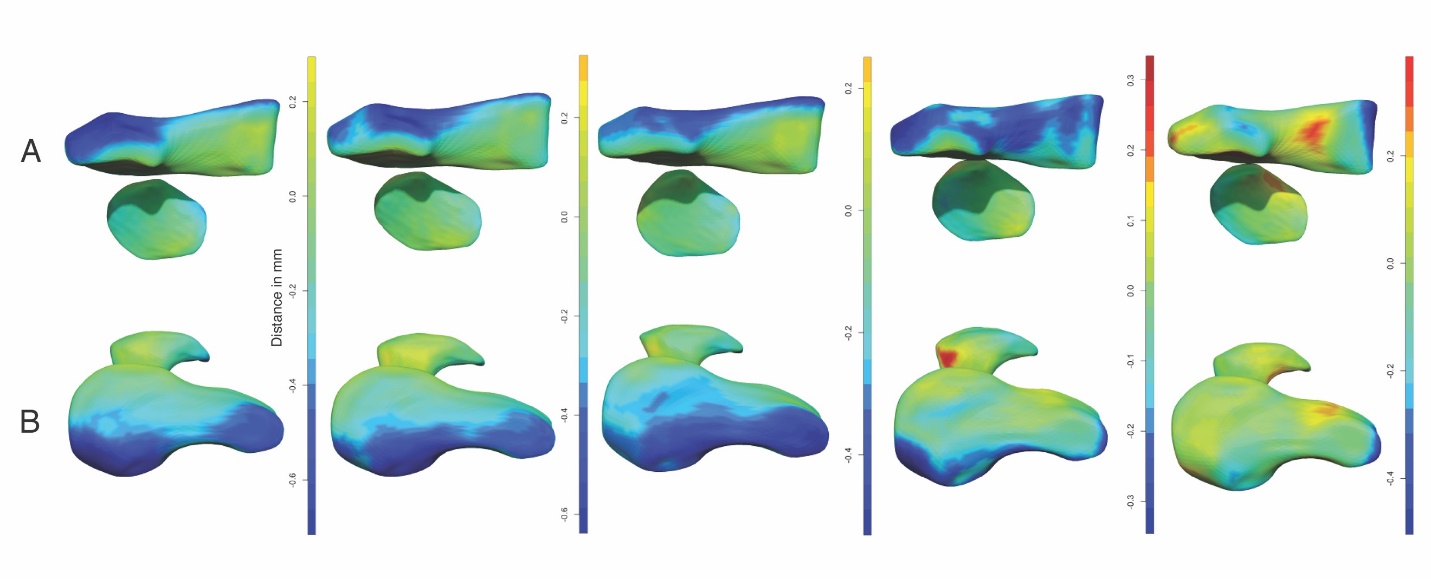


Supplementary Figure 9. Modelled mesh heatmaps showing distances between modelled and original membranous utricle and membranous saccule. A) Lateral view; B) Superior view. The majority of mesh differences lies in the anterolateral shape and position of the utricle. Distances are in mm. Meshes are not to scale.

| **Supplementary Table 1. Study sample. Right three columns indicate if the scan was used in a particular analysis** | | | | | |
| --- | --- | --- | --- | --- | --- |
| **Specimen #** | **Specimen ID** | **Voxel resolution (mm)** | **Bony SPHARM** | **Membranous SPHARM** | **Landmarking** |
| 1 | EmbalmHuman_18p9Upix_130115_axial | 0.009 | No | No | Yes |
| 2 | Human9265L_10Upix_190216_axial | 0.010 | Yes | Yes | Yes |
| 3 | Marcus2_Human_9Os_Laby_240813 Frontal 25Upix | 0.025 | Yes | No | Yes |
| 4 | Marcus2R_Laby_11Upix_060814_axial | 0.011 | Yes | Yes | Yes |
| 5 | Human8081R_2prcnt_7Os_8p3Upix_090414_frontal | 0.008 | No | No | Yes |
| 6 | Marcus_Human_5p5Os_24p7Upix_060713_Frontal | 0.025 | Yes | Yes | Yes |
| 7 | OscarR_11Upix_270914_Frontal | 0.011 | Yes | No | No |
| 8 | Humanly2_9p4Upix_090915_Frontal | 0.009 | Yes | Yes | Yes |
| 9 | Human9265R_10p7Upix_170216_Frontal | 0.011 | Yes | Yes | Yes |
| 10 | 892105L_Human_30hOs_310513_23p4Upix_020613_frontal | 0.023 | Yes | No | Yes |
| 11 | Marcus_Human_3Os_28p4Upix_170613_frontal | 0.028 | Yes | No | Yes |
| 12 | Human8081L_2Prcnt_2Os_9p5Upix_230314_Frontal | 0.010 | No | No | Yes |
| 13 | Human9267L_4Os_10p7Upix_170416_frontal | 0.011 | Yes | No | Yes |

| **Supplementary Table 2. Protocol for cropping the bony and membranous labyrinth for SPHARM analyses** | |
| --- | --- |
| Cut # | Description |
| **Bony External Structure** | |
| 1 | Cut where bony anterior ampulla meets bony vestibule |
| 2 | Cut where bony lateral ampulla meets bony vestibule |
| 3 | Cut along boundary of where the bony posterior ampulla meets bony vestibule |
| 4 | Horizontal cut where common crus meets bony vestibule (in orientation where lateral canal is positioned horizontal and perpendicular to viewer with the anterior bony ampulla overlaying the common crus; termed anterior vestibular horizontal view: AVH) |
| 5 | Cut along boundary of where the slender portion of the lateral canal meets bony vestibule |
| 6 | Horizontal cut at inferior border of spherical recess in AVH |
| **Membranous Internal Structure** | |
| 7 | Cut where membranous anterior ampulla meets membranous utricle |
| 8 | Cut where membranous lateral ampulla meets membranous utricle |
| 9 | Cut where utricular duct meets membranous utricle |
| 10 | Horizontal cut where common crus meets membranous utricle (in orientation where lateral canal is positioned horizontal and perpendicular to viewer with the spherical recess facing the viewer; termed the medial vestibular horizontal view: MVH) |
| 11 | Cut where the slender portion of the lateral semicircular duct meets membranous utricle |
| 12 | Horizontal cut on the membranous labyrinth at the level where the vestibular aqueduct meets bony posterior ampulla in MVH. |
| 13 | Cut where the saccular duct meets membranous saccule |
| 14 | Cut where ductus reuniens meets membranous saccule |
| 15 | Split membranous utricle and membranous saccule into two separate meshes |

| **Supplementary Table 3. Landmarks** | |
| --- | --- |
| **Landmark** | **Description** |
| **Bony Vestibule** | |
| 1 | Posteriormost point of where lateral bony ampulla meets bony vestibule |
| 2 | Superior edge of the posteriormost point on the inferior crista of the supraovalic fossa |
| 3 | Inferior edge of where utricular nerve enters the vestibule (placed along line drawn vertically from anterior bound of the oval window in AVH) |
| 4 | Inferior edge of superiormost point of utricular crest (placed along line drawn vertically from anterior bound of the oval window in AVH)  *Utricular crest is defined here as the ridge formed on the bony labyrinth endocast at the superior border of where the anterior ampullar nerve meets the labyrinth |
| 5 | Point at which the elliptical recess meets the spherical recess along the crista vestibuli in AVH |
| 6 | Inferiormost point where posterior bony ampulla meets the vestibular aqueduct (we term this the aqueductal point) |
| 7 | Anteriormost point of oval window |
| 8 | Posteriormost point of spherical recess |
| 9 | Superiomost point of spherical recess in MVH |
| 10 | Anteroinferiormost point of spherical recess in MVH |
| SemiLM1 | Boundary of where the spherical recess meets the rest of the bony vestibule (19 semilandmarks, resampled, starting at landmark 8, moving superiorly)  *This contour was used instead of complete coverage of the spherical recess since segmentation artifacts and/or variations in the innervation of the saccular macula could alter mesh surface |
| SemiLM2 | Boundary of the oval window (19 semilandmarks, resampled, starting at landmark 7, moving superiorly) |
| **Utricle** | |
| 11 | Posteriormost point of utricular hook inferior edge |
| 12 | Point where utricular ridge meets medial boundary of anterior ampulla |
| 13 | Point where utricular ridge meets membranous labyrinth posteriorly |
| ­­­SemiLM3 | Resampled curve spanning from landmarks 12 to 13 (10 semilandmarks) |
| **Saccule** | |
| 14 | Tip of superolateral projection of saccule |
| 15 | Anterosuperior junction between the saccule and saccular duct |
| 16 | Anteroinferior junction between saccule and ductus reuniens |
| **Utricular macula** | |
| 17 | Posteriormost point of utricular macula |
| 18 | Anteriormost point of the superior edge of curved anterior section of the utricular macula |
| 19 | Medialmost point of the superior edge of curved anterior section of the utricular macula |
| SemiLM4 | Resampled curve along contour of utricular macular surface (19 semilandmarks starting at landmark 17, moving anterolaterally) |
| **Saccular macula** | |
| 20 | Posteriormost point of saccular macula |
| 21 | Posteriormost point of dorsal flap |
| 22 | Anteroinferiormost point of saccular macula |
| SemiLM5 | Resampled curve along contour of saccular macular surface (19 semilandmarks starting at landmark 20, moving inferiorly) |

| **Supplementary Table 4. Justifications for landmark selection** | |
| --- | --- |
| **Landmark** | **Justification** |
| **Bony Vestibule** | |
| 1 | Provides a superolateral boundary to the utricle since the lateral ampulla arises from the utricular walls |
| 2 | Indicates the posterolateral tethering of the ML and utricle |
| 3 | Indicates the inferomedial boundary of the neural substrate of utricular macula |
| 4 | Indicates the superiormost bound of curved anterior sector of utricular macula [1] |
| 5 | Medial boundary of ML attachment |
| 6 | Most reliable attachment site of the ML to the bony vestibular wall [2] |
| 7 | Anterior bound of oval window. Approximates anterolateral bound of bony saccule based on anterior ML attachment [2] |
| 8 | Directly overlies posterior tip of saccular macula |
| 9 | Approximates the medial attachment of the ML directly superior to the saccule |
| 10 | Approximates the anterior and inferior bounds of the spherical recess in its closest proximity to the oval window and basal turn of the cochlea. |
| SemiLM1 | Captures circumference, orientation, and curvature of spherical recess |
| SemiLM2 | Captures circumference, orientation, and curvature of oval window, and thus the positioning of the stapedial footplate. |
| **Utricle** | |
| 11 | Lateral bound of the utricle matching the posterolateral attachment of the ML. Captures lateral extent of utricular hook. |
| 12 | Anterior bound of membranous utricle |
| 13 | Posterior bound of membranous utricle |
| SemiLM3 | Captures curvature of medial membranous utricle |
| **Saccule** | |
| 14 | Superolateral bound of membranous saccule |
| 15 | Posterosuperior bound of membranous saccule |
| 16 | Posteroinferior bound of membranous saccule |
| **Utricular macula** | |
| 17 | Posteriormost point of utricular macula. Matches ML attachment to the inferior crista of the supraovalic fossa |
| 18 | Captures anterolateral upward curvature of the utricular macula |
| 19 | Captures anteromedial upward curvature of the utricular macula [3] |
| SemiLM4 | Captures contour of utricular macula |
| **Saccular macula** | |
| 20 | Posterior bound of saccular macula and underlies landmark 8 |
| 21 | Captures position of dorsal flap of saccular macula |
| 22 | Anteroinferior bound of saccular macula [4] |
| SemiLM5 | Captures contour of saccular macula |

| **Supplementary Table 5. OLS regressions between external bone and internal soft tissue shape. Linear models consist of the primary membranous shape component (PC1) as the explanatory variable and primary bony shape component (PC1) as the response variable.** | | | | | |
| --- | --- | --- | --- | --- | --- |
| **Landmarks included** | **Coefficient** | **Std. Error** | **t** | **R^2^** | **P** |
| **Overall vestibule** |  |  |  |  |  |
| All bone landmarks vs. All membranous landmarks (Set 1; Bone = landmarks 1-10, SemiLMs 1 and 2; Membranous = landmarks 11-22, SemiLMs 3-5) | 0.704 | 0.2165 | 3.251 | 0.47 | **0.00871** |
| All landmarks except SemiLM2 (oval window) (Set 2; Bone = landmarks 1-10, SemiLM1; Membranous = landmarks 11-22, SemiLMs 3-5) | 0.27 | 0.1622 | 1.665 | 0.14 | 0.127 |
| All except SemiLMs (Set 3; Bone = landmarks 1-10; Membranous = landmarks 11-22) | 0.203 | 0.2607 | 0.777 | -0.037 | 0.455 |
| All except SemiLMs and LM 5 (Set 4; Bone = landmarks 1-4, 6-10; Membranous = landmarks 11-22) | 0.246 | 0.2567 | 0.958 | -0.008 | 0.361 |
| All except SemiLMs and LM 5 & 7 (Set 5; Bone = landmarks 1-4, 6, 8-10; Membranous = landmarks 11-22) | -0.011 | 0.2459 | -0.046 | -0.10 | 0.964 |
| **Utricle** |  |  |  |  |  |
| All bone landmarks vs. All membranous landmarks (Set 6; Bone = landmarks 1-6; Membranous = landmarks 11-13, 17-19, SemiLMs 3,4) | -0.468 | 0.1795 | -2.606 | 0.35 | **0.0262** |
| All except SemiLMs (Set 6; Bone = landmarks 1-6; Membranous = landmarks 11-13, 17-19) | 0.443 | 0.208 | 2.128 | 0.2428 | 0.059 |
| **Saccule** |  |  |  |  |  |
| All bone landmarks vs. All membranous landmarks (Set 6; Bone = landmarks 7-10, SemiLMs 1 and 2; Membranous = landmarks 14-16, 20-22, SemiLM5) | 0.969 | 0.233 | 4.15 | 0.60 | **0.002** |
| All landmarks except SemiLM2 (oval window) (Set 6; Bone = landmarks 7-10, SemiLM1; Membranous = landmarks 14-16, 20-22, SemiLM5) | 0.427 | 0.3282 | 1.3 | 0.059 | 0.223 |
| All landmarks except SemiLMs (Set 6; Bone = landmarks 7-10; Membranous = landmarks 14-16, 20-22) | -0.113 | 0.238 | -0.047 | -0.076 | 0.646 |
| All landmarks except SemiLMs and LMs 14-16 (Set 6; Bone = landmarks 7-10; Membranous = landmarks 20-22) | 0.204 | 0.2795 | 0.731 | -0.04 | 0.48 |

| **Supplementary Table 6. OLS regressions between external bone and internal soft tissue centroid sizes. Linear models: Bone centroid size ~ Membranous centroid size.** | | | | | |
| --- | --- | --- | --- | --- | --- |
| **Model** | **Coefficient** | **Std. Error** | **t** | **R^2^** | **P** |
| **Utricle Size (single LMs only)** |  |  |  |  |  |
| log(Bony Utricle 1 centroid size)~log(Membranous Utricle centroid size without macula) | 0.4724 | 0.1139 | 4.149 | 0.6 | **0.00198** |
| log(Bony Utricle 2 centroid size)~log(Membranous Utricle centroid size without macula) | 0.47 | 0.1281 | 3.67 | 0.53 | **0.00432** |
| log(Bony Utricle 3 centroid size)~log(Membranous Utricle centroid size without macula) | 0.4914 | 0.1569 | 3.131 | 0.44 | **0.01067** |
| log(Bony Utricle 1 centroid size)~log(Membranous Utricle + Utricular macula centroid size) | 0.3792 | 0.1275 | 2.975 | 0.42 | **0.0139** |
| log(Bony Utricle 2 centroid size)~log(Membranous Utricle + Utricular macula centroid size) | 0.3974 | 0.1327 | 2.994 | 0.42 | **0.0135** |
| log(Bony Utricle 3 centroid size)~log(Membranous Utricle + Utricular macula centroid size) | 0.3856 | 0.1657 | 2.327 | 0.29 | **0.04228** |
| log(Bony Utricle 2 centroid size)~log(Utricular macula centroid size) | 0.21045 | 0.11879 | 1.772 | 0.16 | 0.107 |
| log(Bony Utricle 1 centroid size)~log(Utricular macula centroid size) | 0.1855 | 0.11641 | 1.593 | 0.12 | 0.142 |
| log(Bony Utricle 3 centroid size)~log(Utricular macula centroid size) | 0.19567 | 0.14022 | 1.395 | 0.08 | 0.193 |
| **Saccule Size (single LMs only)** |  |  |  |  |  |
| log(Bony Saccule 1 centroid size)~log(Saccular macula centroid size) | 0.46053 | 0.12065 | 3.817 | 0.55 | **0.00339** |
| log(Bony Saccule 2 centroid size)~log(Saccular macula centroid size) | 0.34674 | 0.1169 | 2.966 | 0.41 | **0.0141** |
| log(Bony Saccule 2 centroid size)~log(Saccule + Saccular macula centroid size) | 0.3461 | 0.1345 | 2.573 | 0.34 | **0.02775** |
| log(Bony Saccule 1 centroid size)~log(Saccule + Saccular macula centroid size) | 0.3428 | 0.1735 | 1.975 | 0.21 | 0.07645 |
| log(Bony Saccule 2 centroid size)~log(Membranous Saccular centroid size without macula) | 0.0925 | 0.1198 | 0.772 | -0.04 | 0.458 |
| log(Bony Saccule 1 centroid size)~log(Membranous Saccular centroid size without macula) | 0.07009 | 0.14382 | 0.487 | -0.07 | 0.637 |

*Definitions: Bony Utricle 1=landmarks 1-6; Bony Utricle 2=landmarks 2-6; Bony Utricle 3=landmarks 2-5; Membranous Utricle + Utricular macula=landmarks 11-13, 17-19; Membranous utricle centroid size without macula=landmarks 11-13; Utricular macula=landmarks 17-19; Bony Saccule 1=landmarks 7-10; Bony Saccule 2=landmarks 8-10; Saccule + saccular macula=landmarks 14-16, 20-22; Membranous saccule centroid size without macula=landmarks 14-16; Saccular macula=landmarks 20-22.

| **Supplementary Table 7. Angles used to estimate accuracy of bony plane approximation of maculae** | |
| --- | --- |
| Set | Description |
| 1 | Angle between planes fit to landmarked saccular macula and landmarked spherical recess |
| 2 | Angle between plane fit to landmarked saccular macula and plane of best fit of the selected spherical recess |
| 3 | Angle between plane of best fit of selected saccular macula and plane fit to the landmarked spherical recess |
| 4 | Angle between planes of best fit of saccular macula and spherical recess |
| 5 | Angle between planes fit to landmarked utricular macula and bony landmarks 2,4, and 6 |
| 6 | Angle between planes fit to landmarked utricular macula and bony landmarks 2,4, and 5 |
| 7 | Angle between plane of best fit of selected utricular macula and bony landmarks 2,4, and 6 |
| 8 | Angle between plane of best fit of selected utricular macula and bony landmarks 2,4, and 5 |

| **Supplementary Table 8. Results from tests of integration and modularity** | | | |
| --- | --- | --- | --- |
| **Test (10000 permutations)** |  |  |  |
| ***Integration between external bone and internal soft tissue*** | **r-PLS** | **Effect size (Z)** | **P-value** |
| All bone landmarks vs. All membranous landmarks (Set 1; Bone = landmarks 1-10, SemiLMs 1 and 2; Membranous = landmarks 11-22, SemiLMs 3-5) | 0.9503 | 2.408 | **0.0027** |
| All landmarks except SemiLM2 (oval window) (Set 2; Bone = landmarks 1-10, SemiLM1; Membranous = landmarks 11-22, SemiLMs 3-5) | 0.927 | 1.175 | 0.1292 |
| All except SemiLMs (Set 3; Bone = landmarks 1-10; Membranous = landmarks 11-22) | 0.941 | 1.835 | **0.0292** |
| All except SemiLMs and LM 5 (Set 4; Bone = landmarks 1-4, 6-10; Membranous = landmarks 11-22) | 0.939 | 1.892 | **0.0262** |
| All except SemiLMs and LM 5 & 7 (Set 5; Bone = landmarks 1-4, 6, 8-10; Membranous = landmarks 11-22) | 0.9441 | 1.766 | **0.0352** |
|  |  |  |  |
| ***Integration between greater utricle and greater saccule*** |  |  |  |
| All greater utricle landmarks vs. all greater saccule landmarks with landmark 6 as part of utricle (Set 6; Utricle = landmarks 1-6, 11-13, 17-19, SemiLMs 3 and 4; Saccule = landmarks 7-10, 14-16, 20-22, SemiLMs 1, 2, 5) | 0.9508 | 2.339 | **0.0054** |
| All greater utricle landmarks vs. all greater saccule landmarks with landmark 6 as part of saccule (Set 6; Utricle = landmarks 1-5, 11-13, 17-19, SemiLMs 3 and 4; Saccule = landmarks 6-10, 14-16, 20-22, SemiLMs 1, 2, 5) | 0.9507 | 2.328 | **0.005** |
| All greater utricle single landmarks vs. all greater saccule single landmarks with landmark 6 as part of utricle (Set 6; Utricle = landmarks 1-6, 11-13, 17-19; Saccule = landmarks 7-10, 14-16, 20-22) | 0.937 | 1.3199 | 0.097 |
| All greater utricle single landmarks vs. all greater saccule single landmarks with landmark 6 as part of saccule (Set 6; Utricle = landmarks 1-5, 11-13, 17-19; Saccule = landmarks 6-10, 14-16, 20-22) | 0.939 | 1.415 | 0.082 |
|  |  |  |  |
| ***Integration between hard and soft tissue of greater utricle*** |  |  |  |
| All bone landmarks vs. All membranous landmarks (Set 6; Bone = landmarks 1-6; Membranous = landmarks 11-13, 17-19, SemiLMs 3,4) | 0.932 | 2.16 | **0.0093** |
| All except SemiLMs (Set 6; Bone = landmarks 1-6; Membranous = landmarks 11-13, 17-19) | 0.954 | 2.91 | **0.0005** |
|  |  |  |  |
| ***Integration between hard and soft tissue of greater saccule*** |  |  |  |
| All bone landmarks vs. All membranous landmarks (Set 6; Bone = landmarks 7-10, SemiLMs 1 and 2; Membranous = landmarks 14-16, 20-22, SemiLM5) | 0.9446 | 2.387 | **0.0065** |
| All landmarks except SemiLM2 (oval window) (Set 6; Bone = landmarks 7-10, SemiLM1; Membranous = landmarks 14-16, 20-22, SemiLM5) | 0.9255 | 1.12697 | 0.1353 |
| All landmarks except SemiLMs (Set 6; Bone = landmarks 7-10; Membranous = landmarks 14-16, 20-22) | 0.9 | 1.425 | 0.0801 |
| All landmarks except SemiLMs and LMs 14-16 (Set 6; Bone = landmarks 7-10; Membranous = landmarks 20-22) | 0.9388 | 2.708 | **0.0012** |
|  |  |  |  |
| ***Modularity between external bone and internal soft tissue*** | **CR value** | **Effect size** | **P-value** |
| All bone landmarks vs. All membranous landmarks (Set 1; Bone = landmarks 1-10, SemiLMs 1 and 2; Membranous = landmarks 11-22, SemiLMs 3-5) | 0.914 | -8.37 | **0.0001** |
| All landmarks except SemiLM2 (oval window) (Set 2; Bone = landmarks 1-10, SemiLM1; Membranous = landmarks 11-22, SemiLMs 3-5) | 0.914 | -5.2 | **0.0001** |
| All except SemiLMs (Set 3; Bone = landmarks 1-10; Membranous = landmarks 11-22) | 1.006 | -0.465 | 0.31 |
| All except SemiLMs and LM 5 (Set 4; Bone = landmarks 1-4, 6-10; Membranous = landmarks 11-22) | 1.0147 | -0.28 | 0.373 |
| All except SemiLMs and LM 5 & 7 (Set 5; Bone = landmarks 1-4, 6, 8-10; Membranous = landmarks 11-22) | 1.0154 | -0.312 | 0.382 |
| ***Modularity between greater utricle and greater saccule*** |  |  |  |
| All greater utricle landmarks vs. all greater saccule landmarks with landmark 6 as part of utricle (Set 6; Utricle = landmarks 1-6, 11-13, 17-19, SemiLMs 3 and 4; Saccule = landmarks 7-10, 14-16, 20-22, SemiLMs 1, 2, 5) | 0.9242 | -7.29565 | **0.0001** |
| All greater utricle landmarks vs. all greater saccule landmarks with landmark 6 as part of saccule (Set 6; Utricle = landmarks 1-5, 11-13, 17-19, SemiLMs 3 and 4; Saccule = landmarks 6-10, 14-16, 20-22, SemiLMs 1, 2, 5) | 0.9247 | -7.172 | **0.0001** |
| All greater utricle single landmarks vs. all greater saccule single landmarks with landmark 6 as part of utricle (Set 6; Utricle = landmarks 1-6, 11-13, 17-19; Saccule = landmarks 7-10, 14-16, 20-22) | 0.9961 | -0.6996 | 0.237 |
| All greater utricle single landmarks vs. all greater saccule single landmarks with landmark 6 as part of saccule (Set 6; Utricle = landmarks 1-5, 11-13, 17-19; Saccule = landmarks 6-10, 14-16, 20-22) | 0.99803 | -0.646 | 0.253 |
| ***Modularity between hard and soft tissue of greater utricle*** |  |  |  |
| All bone landmarks vs. All membranous landmarks (Set 6; Bone = landmarks 1-6; Membranous = landmarks 11-13, 17-19, SemiLMs 3,4) | 0.992 | -1.197 | 0.117 |
| All except SemiLMs (Set 6; Bone = landmarks 1-6; Membranous = landmarks 11-13, 17-19) | 1.051 | -0.518 | 0.282 |
| ***Modularity between hard and soft tissue of greater saccule*** |  |  |  |
| All bone landmarks vs. All membranous landmarks (Set 6; Bone = landmarks 7-10, SemiLMs 1 and 2; Membranous = landmarks 14-16, 20-22, SemiLM5) | 0.931 | -4.1167 | **0.0002** |
| All landmarks except SemiLM2 (oval window) (Set 6; Bone = landmarks 7-10, SemiLM1; Membranous = landmarks 14-16, 20-22, SemiLM5) | 0.951 | -1.6298 | 0.0575 |
| All landmarks except SemiLMs (Set 6; Bone = landmarks 7-10; Membranous = landmarks 14-16, 20-22) | 0.99934 | -0.03649 | 0.479 |
| All landmarks except SemiLMs and LMs 14-16 (Set 6; Bone = landmarks 7-10; Membranous = landmarks 20-22) | 1.1339 | -0.0074 | 0.5252 |

*See attached video file

Supplementary Video 1. 3d morphometric model of the human otolith system. Part 1) Human left bony labyrinth; Part 2) SPHARM-PDM of mean utricle (teal) and mean saccule (purple) with maculae contours (shown in white) aligned using consensus landmark configuration; Part 3) SPHARM-PDM of mean utricle and mean saccule with maculae contours shown in isolation; Part 4) Aligned SPHARM-PDMs of mean bony vestibule (yellow), mean utricle (teal) and mean saccule (purple).

**References**

1. J. Corvera, C. S. Hallpike, E. H. J. Schuster, A new method for the anatomical reconstruction of the human macular planes. *Acta Otolaryngol* **49**, 4–16 (1958).

2. C. M. Smith, I. S. Curthoys, P. Mukherjee, C. Wong, J. T. Laitman, Three‐dimensional visualization of the human membranous labyrinth: The membrana limitans and its role in vestibular form. *Anat Rec* **305**, 1037–1050 (2022).

3. A. Tribukait, U. Rosenhall, Directional sensitivity of the human macula utriculi based on morphological characteristics. *Audiol Neurootol* **6**, 98–107 (2001).

4. A. Tribukait, U. Rosenhall, B. Österdahl, Morphological characteristics of the human macula sacculi. *Audiology and Neurotology* **10**, 90–96 (2005).
